# On the Coherence of Probabilistic Relational Formalisms

**DOI:** 10.3390/e20040229

**Published:** 2018-03-27

**Authors:** Glauber De Bona, Fabio G. Cozman

**Affiliations:** Escola Politécnica, Universidade de São Paulo, São Paulo 05508-010, Brazil

**Keywords:** relational Bayesian networks, probabilistic relational models, coherence checking

## Abstract

There are several formalisms that enhance Bayesian networks by including relations amongst individuals as modeling primitives. For instance, Probabilistic Relational Models (PRMs) use diagrams and relational databases to represent repetitive Bayesian networks, while Relational Bayesian Networks (RBNs) employ first-order probability formulas with the same purpose. We examine the *coherence checking* problem for those formalisms; that is, the problem of guaranteeing that any grounding of a well-formed set of sentences does produce a valid Bayesian network. This is a novel version of de Finetti’s problem of coherence checking for probabilistic assessments. We show how to reduce the coherence checking problem in relational Bayesian networks to a validity problem in first-order logic augmented with a transitive closure operator and how to combine this logic-based approach with faster, but incomplete algorithms.

## 1. Introduction

Most statistical models are couched so as to guarantee that they specify a single probability measure. For instance, suppose we have *N* independent biased coins, so that heads has probability *p* for each one of them. Then, the probability of a particular configuration of all coins is exactly $p^{n}{\left(1-p\right)}^{N-n}$, where *n* is the number of heads in the configuration. Using de Finetti’s terminology, we can say that the probabilistic assessments and independence assumptions are *coherent* as they are satisfied by a probability distribution [1]. The study of coherence and its consequences has influenced the foundations of probability and statistics, serving as a subjectivist basis for probability theory [2,3], as a broad prescription for statistical practice [4,5] and generally as a bedrock for decision-making and inference [6,7,8].

In this paper, we examine the coherence checking problem for probabilistic models that enhance Bayesian networks with relations and first-order formulas: more precisely, we introduce techniques that allow one to check whether a given relational Bayesian network, or a given probabilistic relational model is guaranteed to specify a probability distribution. Note that “standard” Bayesian networks are, given some intuitive assumptions, guaranteed to be coherent [9,10,11]. The challenge here is to handle models that enlarge Bayesian networks with significant elements of first-order logic; we do so by resorting to logical inference itself as much as possible. In the remainder of this section, we explain the motivation for this study and the basic terminology concerning it, and at the end of this section, we state our goals and our approach in more detail.

To recap, a Bayesian network consists of a directed acyclic graph, where each node is a random variable, and a joint probability distribution over those variables, such that the distribution and the graph satisfy a Markov condition: each random variable is independent of its non-descendants given its parents. (In a directed acyclic graph, node *X* is a *parent* of node *Y* if there is an edge from *X* to *Y*. The set of parents of node *X* is denoted Pa(X). Similarly, we define the children of a node, the descendants of a node, and so on.)

If all random variables $X_{1},\ldots ,X_{n}$ in a Bayesian network are categorical, then the Markov condition implies a factorization:

(1) $$P\left(X_{1}=x_{1},\ldots ,X_{n}=x_{n}\right)=\prod_{i=1}^{n}P\left(X_{i}=x_{i}|\mathrm{Pa}\left(X_{i}\right)=\pi_{i}\right),$$

where $\pi_{i}$ is the projection of $\left\{X_{1}=x_{1},\ldots ,X_{n}=x_{n}\right\}$ on $\mathrm{Pa}(X_{i})$.

Typically, one specifies a Bayesian network by writing down the random variables $X_{1},\ldots ,X_{n}$, drawing the directed acyclic graph, and then settling on probability values $P(X_{i}=x_{i}|\mathrm{Pa}\left(X_{i}\right)=\pi_{i})$, for each $X_{i}$, each $x_{i}$ and each $\pi_{i}$. By following this methodological guideline, one obtains the promised coherence: a unique joint distribution is given by Expression (1).

The following example introduces some useful notation and terminology.

**Figure 1:**
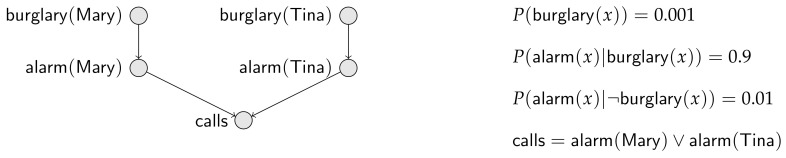
Bayesian network modeling the burglary-alarm-call scenario with Mary and Tina. In the probabilistic assessments (right), the logical variable *x* stands for Mary and for Tina.

**Example** **1.** Consider two neighbors, Mary and Tina. The probability that a house is burglarized is 0.001 in their town. The alarm of a house rings with probability 0.9 given that the house is burglarized and with probability 0.01 given the house is not burglarized. Finally, if either alarm rings, the police are called. This little story, completed with some assumptions of independence, is conveyed by the Bayesian network in Figure 1, where burglary(x) means that the house of x (either Mary or Tina) is burglarized; similarly alarm(x) means that the alarm of x’s house rings; and finally calls just means that the police are called by someone.

In this paper, every random variable is binary with values zero and one, the former meaning “false” and the latter meaning “true”. Furthermore, we often write P(X), where X is a random variable, to mean the event {X=1}, and we often write P(¬X) to mean the event {X=0}.

Note also that we use, whenever appropriate, logical expressions with random variable names, such as alarm(Mary)∨burglary(Tina) to mean the disjunction of the proposition stating that alarm(Mary) is true and the proposition that burglary(Tina) is true. A random variable name has a dual use as a proposition name.

From the Bayesian network in Figure 1, we compute P(alarm(Mary))=0.9×0.001+0.01×0.999=0.01899 and $P\left(\mathrm{calls}\right)=0.01899+0.01899-{\left(0.01899\right)}^{2}$.

**Figure 2:**
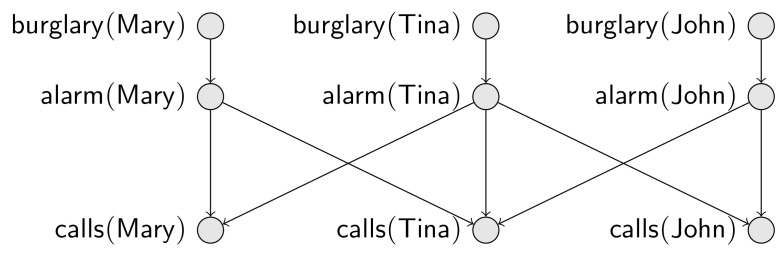
Bayesian network modeling Scenario 3 in Example 2. Probabilistic assessments are just as in Figure 1, except that, for each *x*, calls(x) is the disjunction of its corresponding parents.

Here are some interesting scenarios that enhance the previous example:

**Example** **2.** (Scenario 1) Consider that now we have three people, Mary, Tina and John, all neighbors. We can easily imagine an enlarged Bayesian network, with two added nodes related to John, and a modified definition where calls=alarm(Mary)∨alarm(Tina)∨alarm(John).

(Scenario 2) It is also straightforward to expand our Bayesian network to accommodate n individuals $a_{1},a_{2},\ldots ,a_{n}$, all neighbors. We may even be interested in reasoning about calls without any commitments to a fixed n, where calls is a disjunction over all instances of alarm(x). For instance, we have that $P\left(\neg \mathrm{calls}\right)={\left(1-0.01899\right)}^{n}$; hence, the probability of a call to the police will be larger than half for a city with more than 36 inhabitants. No single Bayesian network allows this sort of “aggregate” inference.

(Scenario 3) Consider a slightly different situation with three people, where: Mary and Tina are neighbors Tina and John are neighbors, but Mary and John are not neighbors. Suppose also that each person may call the police, depending on neighboring alarms. This new situation is codified into the Bayesian network given in Figure 2.

(Scenario 4) Suppose we want to extend Scenario 3 to a town with n people. Without knowing which pairs are neighbors, there is no way we can predict in advance the structure of the resulting Bayesian network. However, we can reason about the possible networks: for instance, we know that each set of n people produces a valid Bayesian network, without any cycles amongst random variables.

There are many other scenarios where probabilistic modeling must handle repetitive patterns such as the ones described in the previous examples, for instance in social network analysis or in processing data in the semantic web [12,13,14]. The need to handle such “very structured” scenarios has led to varied formalisms that extend Bayesian networks with the help of predicates and quantifiers, relational databases, loops and even recursion [15]. Thus, instead of dealing with a random variable *X* at a time, we deal with *parameterized random variables* [16]. We write X(x) to refer to a parameterized random variable that yields a random variable for each fixed *x* in a domain; if we consider individuals *a* and *b* in a domain, we obtain random variables X(a) and X(b).

**Figure 3:**
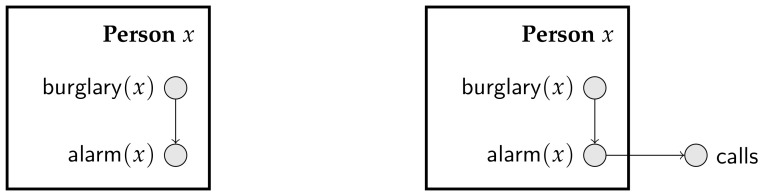
Plate models for Scenario 2 of Example 2; that is, for the burglary-alarm-call scenario where there is a single random variable calls. Left: A partial plate model (without the calls random variable), indicating that parameterized random variables burglary(x) and alarm(x) must be replicated for each person *x*; the domain consists of the set of persons as marked in the top of the plate. Note that each parameterized random variable must be associated with probabilistic assessments; in this case, the relevant ones from Figure 1. Right: A plate model that extends the one on the left by including the random variable calls.

*Plates* offer a popular scheme to manipulate parameterized random variables [17]. A plate is a set of parameterized random variables that share a logical variable, meaning that they are indexed by elements of the same domain. A plate is usually drawn as a rectangle (associated with a domain) containing parameterized random variables. Figure 3 shows simple plate models for the burglary-alarm-call scenario described in Scenario 2 of Example 2.

Plates appeared with the BUGS package, to facilitate the specification of hierarchical models, and have been successful in applications [18]. One restriction of the original plate models in the BUGS package is that a parameterized random variable could not have children outside of its enclosing plate. However, in practice, many plate models violate this restriction. Figure 3 depicts a partial plate model that satisfies the restriction of the original BUGS package (left), and the plate model that violates it (right). Note that as long as the graph consisting of parameterized random variables is acyclic, we know that every Bayesian network generated from the plate model is indeed consistent.

Several other combinations of parameterized random variables and graph-theoretical representations have been proposed, often grouped under the loose term “Probabilistic Relational Model (PRM)” [10,19,20]. Using PRMs, one can associate parameterized random variables with domains, impose constraints on domains and even represent limited forms of recursion [19,21]. A detailed description of PRMs is given in Section 4; for now, it suffices to say that a PRM is specified as a set of “classes” (each class is a set of individuals), where each class is associated with a set of parameterized random variables and additionally by a relational database that gives the relations amongst individuals in classes. The plate model in Figure 3 (left) can be viewed as a diagrammatic representation for a minimalistic PRM, where we have a class Person containing parameterized random variables. Note that such a minimalistic PRM with a single class Person cannot encode Scenario 4 in Example 2, as in that scenario, we have pairs of interacting individuals.

**Figure 4:**
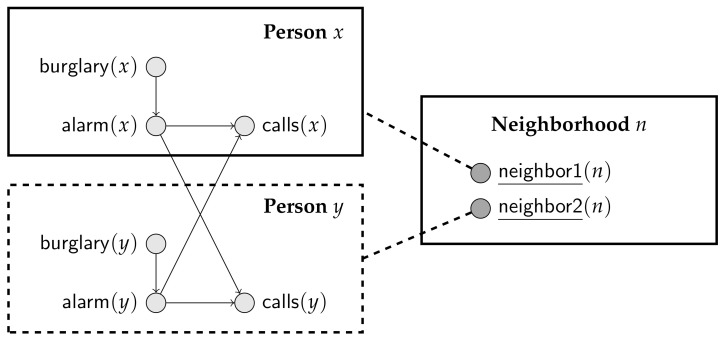
A Probabilistic Relational Model (PRM) for Scenario 4 in Example 2, using a diagrammatic scheme suggested by Getoor et al. [19]. A textual description of this PRM is presented in Section 4.

Suppose that we want a PRM to represent Scenario 4 in Example 2. Now, the class Person must include parameterized random variables burglary, alarm and calls. The challenge is how to indicate which Persons are parents of a particular calls(x). To do so, one possibility is to introduce another class, say Neighborhood, where each element of Neighborhood refers to two elements of Person. In Section 4 we show how the resulting PRM can be specified textually; for now, we want to point out that finding a diagrammatic representing this PRM is not an obvious matter. Using the scheme suggested by Getoor et al. [19], we might draw the diagram in Figure 4. There, we have a class Person, a class Neighborhood and a “shadow” class Person that just indicates the presence of a second Person in any Neighborhood pair. Dealing with all possible PRMs indeed requires a very complex diagrammatic language, where conditional edges and recursion can be expressed [21].

Instead of resorting to diagrams, one may instead focus just on textual languages to specify repetitive Bayesian networks. A very solid formalism that follows this strategy is Jaeger’s Relational Bayesian Networks (RBNs) [22,23]. In RBNs, relations within domains are specified using a first-order syntax as input, returning an output that can be seen as a typical Bayesian network. For instance, using syntax that will be explained later (Section 2), one can describe Scenario 4 in Example 2 with the following RBN:

burglary(x) = 0.001;
alarm(x) = 0.9 * burglary(x) + 0.01 * (1-burglary(x));
calls(x) = NoisyOR { alarm(y) | y; neighbor(x,y) };

One problem that surfaces when we want to use an expressive formalism, such as RBNs or PRMs, is whether a particular model is guaranteed to always produce consistent Bayesian networks. Consider a simple example [19].

**Example** **3.** *Suppose we are modeling genetic relationships using the parameterized random variable gene(x), for any person x. Now, the genetic features of x depend on the genetic features of the mother and the father of x. That is, we want to encode:*

If y and z are such that motherOf(y,x) and fatherOf(z,x) are true, then the probability of gene(x) depends on gene(y) and gene(z).

**Figure 5:**
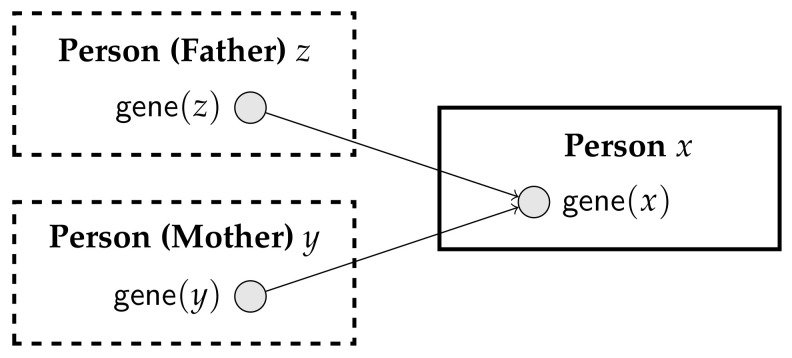
The PRM for the genetic example, as proposed by Getoor et al. [19].

It we try to specify a PRM for this setting, we face a difficulty in that some instances of gene could depend on other instances of the same parameterized random variable. Indeed, consider drawing a diagram for this PRM, using the conventions suggested by Getoor et al. [19]. We would need a class Person, containing parameterized random variable gene, and two shadow classes, one for the father and one for the mother; a fragment of the diagram is depicted in Figure 5. If we could have a Person that appears as the father of his own father, we would have a cycle in the generated Bayesian network. Of course, we know that such a cycle can never be generated because neither the transitive closure of motherOf, nor of fatherOf can contain a cycle. However, just by looking at the diagram, without any background understanding of motherOf and fatherOf, we cannot determine whether coherence is guaranteed.

The possibility that RBNs and PRMs may lead to cyclic (thus inconsistent) Bayesian networks has been noticed before. Jaeger [23] suggested that checking whether an RBN always produces consistent Bayesian networks, for a given class of domains, should be solved by logical inference, being reducible to deciding the validity of a formula from first-order logic augmented with a transitive closure operator. This path has been explored by De Bona and Cozman [24], yielding theoretical results of very high computational complexity. On a different path, Getoor et al. [19] put forward an incomplete, but more intuitive way of ensuring coherence for their PRMs; in fact, assuming that some sets of input relations never form cycles, one can easily identify a few cases where coherence is guaranteed.

Thus, we have arrived at the problems of interest in this paper: Suppose we have an RBN or a PRM. Is it *coherent* in the sense that it can be always satisfied by a probability distribution? Is it *coherent* in the sense that it always produces a unique probability distribution? Such are the questions we address, by exploring a cross-pollination of ideas described in the previous paragraph. In doing so, we bring logical rigor to the problem of coherence of PRMs and present a practical alternative to identifying coherent RBNs.

After formally introducing relational Bayesian networks in Section 2, we review, in Section 3, how its coherence problem can be encoded in first-order logic by employing a transitive closure operator. Section 4 presents PRMs and the standard graph-based approach to their coherence checking. The logical methods developed for the coherence problem of RBNs are adapted to PRMs in Section 5. Conversely, in Section 6, we adapt the graph techniques presented for tackling the coherence of PRMs to the formalism of RBNs.

## 2. Relational Bayesian Networks

In this section, we briefly introduce the formalism of Relational Bayesian Networks (RBNs). We use the version of RBNs presented in [23], as that reference contains a thorough exposition of the topic.

Let *S* and *R* be disjoint sets of relation symbols, called the *predefined relations* and *probabilistic relations*, respectively. We assume that *S* contains the equality symbol =, to be interpreted in the usual way. Each predicate symbol is associated with a positive integer *k*, which is its *arity*. Given a finite domain $D=\{d_{1},\ldots ,d_{n}\}$, if *V* is a set of relation symbols (as *R* or *S*), a *V-structure* D is an interpretation of the symbols in *V* into sets of tuples in *D*. Formally, a *V*-structure D maps each relation symbol v∈V with arity *k* into a subset of $D^{k}$. We denote by ${\mathrm{Mod}}_{D}\left(V\right)$ the set of all *V*-structures over a given finite domain *D*. Given a domain *D*, a v∈V with arity *k* and a tuple $t\in D^{k}$, v(t) is said to be a *ground V-atom*. A *V*-structure D defines truth values for ground atoms: if *v* is mapped to a relation containing *t*, we say that v(t) is *satisfied by* D, which is denoted by D⊨v(t).

Employing the syntax of function-free first-order logic, we can construct formulas using a vocabulary of relations *V*, together with variables, quantifiers and Boolean connectives. We call these *V-formulas*, and their meaning is given by the first-order logic semantics, as usual, through the *V*-structures. We denote by $\varphi (x_{1},\ldots ,x_{m})$ a *V*-formula where $x_{1},\ldots ,x_{k}$ are free variables, in the usual sense. If φ is a *V*-formula and D is a *V*-structure, D⊨φ denotes that φ is satisfied by D.

A *random relational structure model for S and R* is a partial function *P* that takes an *S*-structure D, over some finite domain *D*, and returns a probability distribution $P\left(D\right):{\mathrm{Mod}}_{D}\left(R\right)\to \left[0,1\right]$ over the *R*-structures on the same domain. As an *R*-structure can be seen as total assignment over the ground *R*-atoms, P(D) can be seen as a joint probability distribution over these ground atoms. An example of random relational structure model would be a function $P_{S4}$ in Scenario 4 of Example 2 that receives an *S*-structure of neighbors and returns a joint probability distribution over ground atoms for burglary(·), alarm(·), calls(·). In that scenario, a given configuration D of neighbors, over a given domain *D*, implies a specific Bayesian network whose variables are the ground atoms for burglary(·), alarm(·), calls(·), which encodes a joint probability distribution, $P_{S4}\left(D\right)$, over these variables. If D is the configuration of neighbors from Scenario 4 of Example 2, $P_{S4}\left(D\right)$ would be captured by the Bayesian network in Figure 2.

Relational Bayesian networks provide a way to compactly represent random relational structure models. This is achieved by mapping each *S*-structure into a *ground* Bayesian network that encodes a probability distribution over *R*-structures. To begin, this ground Bayesian network has nodes representing r(t) (ground atoms), for each r∈R and $t\in D^{k}$, where *k* is the arity of *r*. Thus, given the domain *D* of the input *S*-structure, the nodes in the corresponding Bayesian network are already determined. To define the arcs and parameters of the Bayesian network associated with an arbitrary *S*-structure, relational Bayesian networks employ their central notion of *probability formula*.

Probability formulas are syntactical constructions intended to link the probability of a ground atom r(t) to the probabilities of other ground atoms $r^{\prime }\left(t^{\prime }\right)$, according to the *S*-structure. Once an *R*-structure and an *S*-structure are fixed, then for elements $t_{1},\ldots ,t_{k}$ in the domain *D*, a probability formula $F(t_{1},\ldots ,t_{k})$ should evaluate to a number in [0,1].

The definition of probability formulas makes use of *combination functions*, which are functions from finite multi-sets over the interval [0,1] to numbers in the same interval. We use {|·|} to denote multi-sets. For instance, NoisyOR is a combination function such that, if $c_{1},\ldots ,c_{n}\in \left[0,1\right]$, NoisyOR$\left\{\;\right|c_{1},\ldots ,c_{n}\left|\;\right\}=1-\prod_{i=1}^{n}\left(1-c_{i}\right)$.

**Definition** **1.** Given disjoint sets *S* and *R* of relation symbols and a tuple *x* of *k* variables, F(x) is a *(S,R)-probability formula* if:

- (constants) F(x)=c for a c∈[0,1];
- (indicator functions) F(x)=r(x) for an r∈R with arity *k*;
- (convex combinations) $F\left(x\right)=F_{1}\left(x\right)F_{2}\left(x\right)+\left(1-F_{1}\left(x\right)\right)F_{3}\left(x\right)$, where $F_{1}\left(x\right),F_{2}\left(x\right),F_{3}\left(x\right)$ are probability formulas, or;
- (combination functions) $F\left(x\right)=\mathrm{comb}\{\;|F_{1}\left(x,y\right),\ldots ,F_{m}\left(x,y\right)|y;\varphi \left(x,y\right)|\;\}$, where comb is a combination function, $F_{1}\left(x,y\right),\ldots ,F_{m}\left(x,y\right)$ are probability formulas, *y* is a tuple of variables and φ(x,y) is an *S*-formula.

Relational Bayesian networks associate a probability formula $F_{r⋆}\left(x\right)$ to each probabilistic relation r⋆∈R, where *x* is a tuple of *k* variables, with *k* the arity of r⋆:

**Definition** **2.** Given disjoint sets of relation symbols *S* and *R*, the predefined and probabilistic relations, a *relational Bayesian network* is a set $\Phi =\{F_{r}\left(x\right)|r\in R\}$, where each $F_{r}\left(x\right)$ is a (*S*,*R*)-probability formula.

To have an idea of how probability formulas work, consider a fixed *S*-structure $D_{S}$ over a domain *D*. Then, an *R*-structure $D_{R}$ over *D* entails a numeric value for each *ground probability formula* $F_{r⋆}\left(t\right)$, denoted by $F_{r⋆}\left(t\right)\left[D_{R}\right]$, where *t* is tuple of elements in *D*. This is done inductively, by initially defining $r\left(t\right)\left[D_{R}\right]=1$ if $D_{R}⊨r\left(t\right)$, and $r\left(t\right)\left[D_{R}\right]=0$ otherwise, for each r(t), for all r∈R. If $F_{r⋆}\left(x\right)=c$, then $F_{r⋆}\left(t\right)\left[D_{R}\right]=c$, for any tuple *t*. The numeric value of $F_{r⋆}\left(t\right)\left[D_{R}\right]$ for probability formulas that are convex combinations or combination function will require the evaluation of its subformulas $F_{i}$, which recursively end at the evaluation of ground atoms r(t) or constants *c*. As the set of ground atoms whose evaluation is needed to compute $F_{r⋆}\left(t\right)\left[D_{R}\right]$ depends only on the *S*-structure $D_{S}$, and not on $D_{R}$, it is denoted by $\alpha (F_{r⋆}\left(x\right),t,D_{S})$ and can be defined recursively:

- $\alpha (c,t,D_{S})=⌀$;
- $\alpha \left(r\left(x\right),t,D_{S}\right)=\left\{r\left(t\right)\right\}$;
- $\alpha \left(F_{1}\left(x\right)F_{2}\left(x\right)+\left(1-F_{1}\left(x\right)\right)F_{3}\left(x\right),t,D_{S}\right)=\overset{3}{\underset{i=1}{⋃}}\alpha \left(F_{i}\left(x\right),t,D_{S}\right)$;
- $\alpha (\mathrm{comb}\{\;|F_{1}\left(x,y\right),\ldots ,F_{m}\left(x,y\right)|y;\varphi \left(x,y\right)|\;\},t,D_{S})$ is given by:
  $$\underset{t^{\prime }\;s.t.\;D_{S}⊨\varphi \left(t,t^{\prime }\right)}{⋃}\;\overset{m}{\underset{i=1}{⋃}}\alpha \left(F_{i}\left(x,y\right),\left(t,t^{\prime }\right),D_{S}\right).$$
  Here, $\left(t,t^{\prime }\right)$ denotes the concatenation of the tuples *t* and $t^{\prime }$.

For a given *S*-structure $D_{S}$, we can define a dependency relation between the nodes r(t) and $r^{\prime }\left(t^{\prime }\right)$ in the Bayesian network via the probability formulas $F_{r}$ and $F_{r^{\prime }}$ by employing the corresponding α(·,·,·). Intuitively, $\alpha (F_{r}\left(x\right),t,D_{S})$ contains the ground atoms $r^{\prime }\left(t^{\prime }\right)$ whose truth value in a structure $D_{R}$ determines the value of $F_{r}\left(t\right)$, which is meant to be the probability of r(t). That is, $\alpha (F_{r}\left(x\right),t,D_{S})$ contains the parents of r(t).

**Definition** **3.** *Relation ⪯, over ground R-atoms, is defined as follows:*

$$r\left(t\right)⪯r^{\prime }\left(t^{\prime }\right)\;iff\;r^{\prime }\left(t^{\prime }\right)\in \alpha \left(F_{r}\left(x\right),t,D_{S}\right).$$

When this relation is acyclic, a relational Bayesian network $\Phi =\{F_{r}|r\in R\}$ defines, for a given *S*-structure $D_{S}$ over a finite domain *D*, a probability distribution over the *R*-structures $D_{R}$ over *D* via:

$$P_{D_{S}}^{\Phi }\left(D_{R}\right)=\prod_{r\in R}\;\;\prod_{t,D_{R}⊨r\left(t\right)}F_{r}\left(t\right)\left[D_{R}\right]\prod_{t,D_{R}⊭r\left(t\right)}\left(1-F_{r}\left(t\right)\left[D_{R}\right]\right)$$

**Example** **4.** *Scenario 4 of Example 2: We can define a relational Bayesian network that returns the corresponding Bayesian network for each number and configuration of neighbors. Let S={neighbor(·,·)} and R={burglary(·),alarm(·),calls(·)}. We assume that the relation neighbor is reflexive and symmetrical. For each relation in R, we associate a probability formula, forming the relational Bayesian network* Φ*:*

- $F_{\mathrm{burglary}}\left(x\right)=0.001$; a constant;
- $F_{\mathrm{alarm}}\left(x\right)=0.9\mathrm{burglary}\left(x\right)+0.01\left(1-\mathrm{burglary}\left(x\right)\right)$; a convex combination;
- $F_{\mathrm{call}}\left(x\right)=$ NoisyOR{|alarm(y)∣y;neighbor(x,y)|}*; a combination function.*

*Note that, if $F_{1}\left(x\right)$ and $F_{2}\left(x\right)$ are probability formulas, then $1-F_{1}\left(x\right)$ and $F_{1}\left(x\right)F_{2}\left(x\right)$ are convex combinations and, thus, probability formulas. As the inputs of the* NoisyOR *above are in {0,1}, the combination function actually works like a disjunction.*

Given an S-structure $D_{S}$ over a domain D, *Φ* determines a joint probability distribution over the ground R-atoms, via a Bayesian network. If we take an S-structure $D_{S}$ over a domain $D=\{d_{1},d_{2},d_{3}\}$ such that $D_{S}⊨\mathrm{neighbor}\left(d_{1},d_{2}\right)\wedge \mathrm{neighbor}\left(d_{2},d_{3}\right)$, but $D_{S}⊭\mathrm{neighbor}\left(d_{1},d_{3}\right)$, the resulting $P_{D_{S}}^{\Phi }$ is the model for Scenario 3 in Example 2, whose Bayesian network is given in Figure 2.

## 3. The Coherence Problem for RBNs

It may happen for a relational Bayesian network Φ that some *S*-structures yield a cyclic dependency relation ⪯. When the relation ⪯ is cyclic for an *S*-structure, no probability distribution is defined over the *R*-structures. In such a case, we say Φ is *incoherent* for that *S*-structure. This notion can be generalized to a class of *S*-structures S, so that we say that Φ is *coherent* for S iff the resulting relation ⪯ is acyclic for each *S*-structure in S. To know whether a relational Bayesian network is coherent for a given class of *S*-structures is precisely one of the problems we are interested in this work.

In order to reason about the relation between a class of *S*-structures and the coherence of a relational Bayesian network Φ for it, we need to formally represent these concepts. To define a class of *S*-structures, note that they can be seen as first-order structures over which *S*-formulas are interpreted. That is, an *S*-formula defines the set of *S*-structures satisfying it. If φ is a closed *S*-formula (without free variables), we say that [[φ]] is the set of *S*-structures $D_{S}$ such that $D_{S}⊨\varphi$. We denote by $\theta_{S}$ an *S*-formula satisfied exactly by the *S*-structures in a given class S; that is, $[\left[\theta_{S}\right]]=S$.

To encode the coherence of Φ, we need to encode the acyclicity of the dependency relation ⪯ resulting from an *S*-structure. Ideally, we would like to have a (first-order) *S*-formula, say $\psi_{\Phi }$, that would be true only for *S*-structures yielding acyclic dependency relations ⪯. If that formula were available, a decision about the coherence of Φ for the class S would be reduced to a decision about the validity of the first-order formula $\theta_{S}\to \psi_{\Phi }$: When the formula is valid, then every *S*-structure in the class S guarantees that the resulting dependency relation ⪯ for Φ is acyclic; hence, Φ is coherent for S; otherwise, there is an *S*-structure in S yielding a cyclic dependency relation ⪯ for Φ. Note that for *S*-formulas, only *S*-structures matter, and we could ignore any relation not in *S*. To be precise, if a first-order structure D falsifies $\theta_{S}\to \psi_{\Phi }$, then there is an *S*-structure $D_{S}$ (formed by ignoring non-S relations) falsifying it.

Alas, to encode cycles in a graph, one needs to encode the notion of path, which is the transitive closure of a relation encoding arcs. It is a well-known fact that first-order logic cannot express transitivity. To circumvent that, we can add a (strict) transitive closure operator to the logic, arriving at the so-called transitive closure logics, as described for instance in [25].

This approach was first proposed by Jaeger [23], who assumed one could write down the *S*-formula $\psi_{\Phi }$ by employing a transitive closure operator. He conjectured that with some restrictions on the arity of the relations in *S* and *R*, one could hope to obtain a formula $\theta_{S}\to \psi_{\Phi }$ that is decidable. Nevertheless, no hint was provided as to how to construct such a formula, or as to its general shape. A major difficulty is that, if an *S*-structure D satisfying $\theta_{S}$ has domain $D=\{d_{1},\ldots ,d_{n}\}$, the size of the resulting Bayesian network is typically greater than *n*, with one node per ground atom, so a cycle can also contain more nodes than *n*. There seems to be no direct way of employing the transitive closure operator to devise a formula $\neg \psi_{\Phi }$ that encodes cycles with more than *n* nodes and that is to be satisfied by some structures D over a domain with only *n* elements. In the next sections, we review a technique (introduced by the authors in [24]) to encode $\psi_{\Phi }$ for an augmented domain, through an auxiliary formula whose satisfying structures will represent both the *S*-structure and the resulting ground Bayesian network. Afterwards, we adapt the formula $\theta_{S}$ accordingly.

### 3.1. Encoding the Structure of the Ground Bayesian Network

Our idea to construct a formula $\psi_{\Phi }$, for a given relational Bayesian network Φ, is first to find a first-order *V*-formula $B_{\Phi }$, for some vocabulary *V* containing *S*, that is satisfiable only by *V*-structures that encode both an *S*-structure $D_{S}$ and the structure of the ground Bayesian network resulting from it. These *V*-structures should contain, besides an *S*-structure $D_{S}$, an element for each node in the ground Bayesian network and a relation capturing its arcs. Then, we can use a transitive closure operator to define the existence of paths (and cycles) via arcs, for enforcing acyclicity by negating the existence of a cycle.

Suppose we have two disjoint vocabularies *S* and $R=\{r_{1},\ldots ,r_{m}\}$ of predefined and probabilistic relations, respectively. We use a(v) to denote the arity of a relation *v*. Consider a relational Bayesian network $\Phi =\{F_{r}\left(x\right)|r\in R\}$, where each $F_{r}\left(x\right)$ is a (*S*,*R*)-probability formula. Let D be a *V*-structure satisfying $B_{\Phi }$. We want D to be defined over a bipartite domain $D=D_{S}\cup D_{B}$, where $D_{S}$ is used to represent an *S*-structure $D_{S}$ and $D_{B}=D\setminus D_{S}$ is the domain where the structure of the resulting ground Bayesian network is encoded. We overload names by including in *V* a unary predicate $D_{S}\left(\cdot \right)$ that shall be true for all and only the elements in $D_{S}$. The structure D shall represent the structure of the ground Bayesian network $B_{\Phi }\left(D_{S}\right)$, over the elements of $D_{B}$, that is induced by the *S*-structure $D_{S}$ codified in $D_{S}$. In order to accomplish that, D must have an element in $D_{B}$ for each ground atom over the domain $D_{S}$. Furthermore, the *V*-structure D must interpret a relation, say Parent(·,·), over $D_{B}$ according to the arcs of the Bayesian network $B_{\Phi }\left(D_{S}\right)$.

Firstly, we need to define a vocabulary *V* that includes the predefined relations in *S* and contains the unary predicate $D_{S}$ (recall that the equality symbol (=) is included in *S*). Furthermore, *V* must contain a binary relation Parent to represent the arcs of the ground Bayesian network. As auxiliary relations for defining Parent, we will need a relation $Dep_{i}^{j}$, for each pair $r_{i},r_{j}\in R$, whose arity is $a\left(r_{i}\right)+a\left(r_{j}\right)$. For elements in $D_{B}$ to represent ground atoms $r(t_{1},\ldots ,t_{n})$, we use relations to associate elements in $D_{B}$ to relations *r* and to tuples $\langle t_{1},\ldots ,t_{n}\rangle$. For each relation $r_{i}\in R$, we have a unary relation ${\bar{r}}_{i}\in V$, where ${\bar{r}}_{i}\left(x\right)$ is intended to mean that the element $x\in D_{B}$ represents a ground atom of the form $r_{i}\left(\cdot \right)$. As for the tuples, recall that each $t_{i}$ represents an element in the set $D_{S}$ over which the *S*-structure $D_{S}$ is codified. Hence, we insert in *V* binary relations $t_{i}$ for every $1\leq i\leq \max_{i}\;a\left(r_{i}\right)$, such that $t_{i}\left(x,y\right)$ should be true iff the element $x\in D_{B}$ corresponds to a ground atom $r(t_{1},\ldots ,t_{k})$ where $t_{i}=y$, for a $y\in D_{S}$ and some r∈R.

To save notation, we use $R_{i}\left(x,y_{1},\ldots ,y_{k}\right)$ to denote ${\bar{r}}_{i}\left(x\right)\wedge t_{1}\left(x,y_{1}\right)\wedge \ldots \wedge t_{k}\left(x,y_{k}\right)$ henceforth, meaning the element *x* in the domain represents the ground atom $r_{i}\left(y_{1},\ldots ,y_{k}\right)$, where $a(r_{i})=k$.

Now, we proceed to list, step-by-step, the set of conjuncts required in $\psi_{\Phi }$, together with their meaning, for the *V*-structure D in $\left[\left[\psi_{\Phi }\right]\right]$ to hold the desired properties. To illustrate the construction, each set of conjuncts is followed by an example based on the RBN in Example 4, possibly given in an equivalent form for clarity.

We have to ensure that the elements in $D_{B}$ correspond exactly to the ground atoms in the ground Bayesian network $B_{\Phi }\left(D_{S}\right)$.

- Each element in $D_{B}=D\setminus D_{S}$ should correspond to a ground atom for some $r_{i}\in R$. Hence, we have the formula:
  (2) $$\forall x\neg D_{S}\left(x\right)\to \overset{m}{\underset{i=1}{⋁}}{\bar{r}}_{i}\left(x\right).$$
  $$\forall x\neg D_{S}\left(x\right)\to \bar{\mathrm{burglary}}\left(x\right)\vee \bar{\mathrm{alarm}}\left(x\right)\vee \bar{\mathrm{calls}}\left(x\right).$$
- No element may correspond to ground atoms for two different $r_{i}\in R$. Therefore, the formula below is introduced:
  (3) $$\forall x\overset{i\neq j}{\underset{1\leq i,j\leq m}{⋀}}\left(\neg {\bar{r}}_{i}\left(x\right)\vee \neg {\bar{r}}_{j}\left(x\right)\right).$$
  $$\forall x\left(\neg \bar{\mathrm{burglary}}\left(x\right)\vee \neg \bar{\mathrm{alarm}}\left(x\right)\right)\wedge \left(\neg \bar{\mathrm{burglary}}\left(x\right)\vee \neg \bar{\mathrm{calls}}\left(x\right)\right)\wedge \left(\neg \bar{\mathrm{alarm}}\left(x\right)\vee \neg \bar{\mathrm{calls}}\left(x\right)\right).$$
- Each element corresponding to a ground atom should correspond to exactly one tuple. To achieve that, let $k=\max_{j}a\left(r_{j}\right)$, and introduce the formula below:
  (4) $$\forall x\forall y\forall z\overset{k}{\underset{j=1}{⋀}}\left(t_{j}\left(x,y\right)\wedge t_{j}\left(x,z\right)\to y=z\right).$$
  $$\forall x\forall y\forall z(t_{1}\left(x,y\right)\wedge t_{1}\left(x,z\right)\to y=z.$$
- Each element corresponding to a ground atom for a $r_{i}\in R$ should be linked a to tuple with arity $a(r_{i})$. Thus, let $k=\max_{j}a\left(r_{j}\right)$, and introduce the formula below for each $r_{i}\in R$:
  (5) $$\forall x{\bar{r}}_{i}\left(x\right)\;\to \;\left(\exists y_{1}\ldots \exists y_{a(r_{i})}R_{i}\left(x,y_{1},\ldots ,y_{a(r_{i})}\right)\wedge \forall z\neg t_{a(r_{i})+1}\left(x,z\right)\wedge \cdots \wedge \neg t_{k}\left(x,z\right)\right).$$
  $$\forall x\bar{\mathrm{burglary}}\left(x\right)\;\to \;\left(\exists yt_{1}\left(x,y\right)\right);\;\forall x\bar{\mathrm{alarm}}\left(x\right)\;\to \;\left(\exists yt_{1}\left(x,y\right)\right);\;\forall x\bar{\mathrm{calls}}\left(x\right)\;\to \;\left(\exists yt_{1}\left(x,y\right)\right).$$
- Only elements in $D_{B}=D\setminus D_{S}$ should correspond to ground atoms. This is enforced by the following formula, where $k=\max_{i}a\left(r_{i}\right)$:
  (6) $$\forall yD_{S}\left(y\right)\to \left(\overset{m}{\underset{i=1}{⋀}}\neg {\bar{r}}_{i}\left(y\right)\wedge \forall x\overset{k}{\underset{j=1}{⋀}}\neg t_{j}\left(y,x\right)\right).$$
  $$\forall yD_{S}\left(y\right)\to \left(\neg \bar{\mathrm{burglary}}\left(y\right)\wedge \neg \bar{\mathrm{alarm}}\left(y\right)\wedge \neg \bar{\mathrm{calls}}\left(y\right)\wedge \forall x\neg t_{1}\left(y,x\right)\right).$$
- Each ground atom must be represented by at least one element (in $D_{B}=D\setminus D_{S}$). Therefore, for each $r_{i}\in R$, with $a(r_{i})=k$, we need a formula:
  (7) $$\forall y_{1}\ldots \forall y_{k}D_{S}\left(y_{1}\right)\wedge \cdots \wedge D_{S}\left(y_{k}\right)\to \exists xR_{i}\left(x,y_{1},\ldots ,y_{k}\right).$$
  $$\forall yD_{S}\left(y\right)\to \left(\exists x_{1}\bar{\mathrm{burglary}}\left(x_{1}\right)\wedge t_{1}\left(y,x_{1}\right)\right);\mathrm{same}\mathrm{for}\bar{\mathrm{alarm}}\;\mathrm{and}\;\bar{\mathrm{calls}}.$$
  These formulas enforce that each ground atom r(t) is represented by an element *x* that is in $D_{B}$, due to the formula (6).
- No ground atom can be represented by two different elements. Hence, for each $r_{i}\in R$, with $a(r_{i})=k$, we introduce a formula:
  (8) $$\forall y_{1},\ldots \forall y_{k}\forall x\forall zR_{i}\left(x,y_{1},\ldots ,y_{k}\right)\wedge R_{i}\left(z,y_{1},\ldots ,y_{k}\right)\to x=z.$$
  $$\forall y\forall x\forall z\bar{\mathrm{burglary}}\left(x\right)\wedge t_{1}\left(y,x\right)\wedge \bar{\mathrm{burglary}}\left(z\right)\wedge t_{1}\left(z,y\right)\to x=z;\mathrm{same}\mathrm{for}\bar{\mathrm{alarm}}\;\mathrm{and}\;\bar{\mathrm{calls}}.$$

The conjunction of all formulas in (2)–(8) is satisfied only by structures D over the domain $D=D_{S}\cup D_{B}$ such that there is a bijection between $D_{B}$ and the set of all possible ground atoms {r(t)∣ for some r∈R and $t\in D_{S}^{a(r)}\}$. Now, we can put the arcs over these nodes to complete the structure of the ground Bayesian network $B_{\Phi }\left(D_{S}\right)$.

The binary relation Parent must hold only between elements in the domain *D* representing ground atoms r(t) and $r^{\prime }\left(t^{\prime }\right)$ such that $r\left(t\right)⪯r^{\prime }\left(t^{\prime }\right)$. Recall that the dependency relation ⪯ is determined by the *S*-structure $D_{S}$. While the ground atoms represented in $D_{B}$, for a fixed *R*, are determined by the size of $D_{S}$ by itself, the relation Parent between them depends also on the *S*-formulas that hold for the *S*-structure $D_{S}$. We want these *S*-structures to be specified by D over $D_{S}$ only, not over $D_{B}$. To ensure this, we use the following group of formulas:

- For all s∈S, consider the formula below, where a(s)=k:
  (9) $$\forall y_{1}\ldots \forall y_{k}\;s\left(y_{1},\ldots ,y_{k}\right)\to D_{S}\left(y_{1}\right)\wedge \cdots \wedge D_{S}\left(y_{k}\right).$$
  $$\forall y_{1}\forall y_{2}\;\mathrm{neighbor}\left(y_{1},y_{2}\right)\to D_{S}\left(y_{1}\right)\wedge D_{S}\left(y_{2}\right).$$

The formula above forces that s(t), for any s∈S, can be true only for tuples $t\in D_{S}^{a(s)}$.

For a known *S*-structure $D_{S}$, it is straightforward to determine which ground atoms $r^{\prime }\left(t^{\prime }\right)$ are the parents of r(t) in the ground Bayesian network $B_{\Phi }\left(D_{S}\right)$. One can use recursively the definition of the set of parents $\alpha (F_{r}\left(x\right),t,D_{S})$ given in Section 2. Nonetheless, with an unknown *S*-structure $D_{S}$ specified in D over $D_{S}$, the situation is a bit trickier. The idea is to construct, for each pair $r_{i}\left(t\right)$ and $r_{j}\left(t^{\prime }\right)$, an *S*-formula $Dep_{i}^{j}\left(t,t^{\prime }\right)$ that is true iff $r_{i}\left(t\right)⪯r_{j}\left(t^{\prime }\right)$ for the $D_{S}$ encoded in D. To define $Dep_{i}^{j}\left(t,t^{\prime }\right)$, we employ auxiliary formulas $C_{F(t)}^{r^{\prime }\left(t^{\prime }\right)}$, for a ground probability formula F(t) and a ground atom $r^{\prime }\left(t^{\prime }\right)$, that will be an *S*-formula that is satisfied by D iff $r^{\prime }\left(t^{\prime }\right)\in \alpha \left(F\left(x\right),t,S\right)$. We define $C_{F(t)}^{r^{\prime }\left(t^{\prime }\right)}$ recursively, starting from the base cases.

- If F(t)=c, for a c∈[0,1], then $C_{F(t)}^{r^{\prime }\left(t^{\prime }\right)}=⊥;\;e.g.,C_{F_{\mathrm{burglary}}\left(t\right)}^{\mathrm{alarm}(t^{\prime })}=⊥$.
- If $F\left(t\right)=r^{\prime \prime }\left(t\right)$, then $C_{F(t)}^{r^{\prime }\left(t^{\prime }\right)}=\left(t^{\prime }=t\right)$ if $r^{\prime }=r^{\prime \prime }$; and $C_{F(t)}^{r^{\prime }\left(t^{\prime }\right)}=⊥$ otherwise;
  $e.g.,C_{F_{\mathrm{burglary}}\left(t\right)}^{\mathrm{burglary}(t^{\prime })}=\left(t=t^{\prime }\right)$ and $C_{F_{\mathrm{burglary}}\left(t\right)}^{\mathrm{calls}(t^{\prime })}=⊥$.

Above, $\left(t^{\prime }=t\right)$ is a short form for $\left(t_{1}^{\prime }=t_{1}\right)\wedge \cdots \wedge \left(t_{k}^{\prime }=t_{k}\right)$, where *k* is the arity of *t*. These base cases are in line with the recursive definition of α(F(x),t,S) presented in Section 2. The third case is also straightforward:

- If $F\left(t\right)=F_{1}\left(t\right)F_{2}\left(t\right)+\left(1-F_{1}\left(t\right)\right)F_{3}\left(t\right)$, then $C_{F(t)}^{r^{\prime }\left(t^{\prime }\right)}=\overset{3}{\underset{i=1}{⋁}}C_{F_{i}\left(t\right)}^{r^{\prime }\left(t^{\prime }\right)}$.
  $C_{F_{\mathrm{alarm}}\left(t\right)}^{\mathrm{burglary}(t^{\prime })}=C_{F_{\mathrm{burglary}}\left(t\right)}^{\mathrm{burglary}(t^{\prime })}\vee C_{0.9}^{\mathrm{burglary}(t^{\prime })}\vee C_{0.01}^{\mathrm{burglary}(t^{\prime })}=\left(t=t^{\prime }\right)\vee ⊥\vee ⊥$

In other words, the computation of $F\left(t\right)\left[D_{R}\right]$ depends on $r^{\prime }\left(t^{\prime }\right)\left[D_{R}\right]$, for some $D_{R}$, if the computation of some $F_{i}\left(t\right)\left[D_{R}\right]$, for 1≤i≤3, depends on $r^{\prime }\left(t^{\prime }\right)\left[D_{R}\right]$.

The more elaborated case happens when F(x) is a combination function, for which there is an *S*-formula involved. Recall that if $F\left(x\right)=\mathrm{comb}\{\;|F_{1}\left(x,y\right),\ldots ,F_{m}\left(x,y\right)|y;\varphi \left(x,y\right)|\;\}$, then the parents of F(t) are given by $\underset{t^{\prime },D_{S}⊨\varphi \left(t,t^{\prime }\right)}{⋃}\overset{m}{\underset{i=1}{⋃}}\alpha \left(F_{i}\left(x,y\right),\left(t,t^{\prime }\right),D_{S}\right)$. Thus, to recursively define $C_{F(t)}^{r^{\prime }\left(t^{\prime }\right)}$, we need an *S*-formula that is satisfied by an *S*-structure $D_{S}$ iff:

$$r^{\prime }\left(t^{\prime }\right)\in \underset{t^{⋆},D_{S}⊨\varphi \left(t,t^{⋆}\right)}{⋃}\overset{m}{\underset{i=1}{⋃}}\alpha \left(F_{i}\left(x,y\right),\left(t,t^{⋆}\right),D_{S}\right).$$

The inner union is analogous to the definition of $C_{F(t)}^{r^{\prime }\left(t^{\prime }\right)}$ for convex combinations. However, to cope with any $t^{⋆}$ such that $D_{S}⊨\varphi \left(t,t^{⋆}\right)$, we need an existential quantification:

- If $F\left(x\right)=\mathrm{comb}\{\;|F_{1}\left(x,y\right),\ldots ,F_{m}\left(x,y\right)|y;\varphi \left(x,y\right)|\;\}$, then we have that:
  $$C_{F(t)}^{r^{\prime }\left(t^{\prime }\right)}=\exists t^{⋆}\varphi \left(t,t^{⋆}\right)\wedge \overset{m}{\underset{i=1}{⋁}}C_{F_{i}\left(t,t^{⋆}\right)}^{r^{\prime }\left(t^{\prime }\right)}.$$
  $$C_{F_{\mathrm{calls}}\left(t\right)}^{\mathrm{alarm}(t^{\prime })}=\exists t^{⋆}\mathrm{neighbor}\left(t,t^{⋆}\right)\wedge C_{F_{\mathrm{alarm}}\left(t^{⋆}\right)}^{\mathrm{alarm}(t^{\prime })}=\exists t^{⋆}\mathrm{neighbor}\left(t,t^{⋆}\right)\wedge \left(t^{⋆}=t^{\prime }\right)$$

Now, we can employ the formulas $C_{F(t)}^{r^{\prime }\left(t^{\prime }\right)}$ to define the truth value of the ground relation $Dep_{i}^{j}\left(t,t^{\prime }\right)$, that codifies when $r_{i}\left(t\right)⪯r_{j}\left(t^{\prime }\right)$.

- For each pair $r_{i},r_{j}\in R$, with $a(r_{i})=k$ and $a\left(r_{j}\right)=k^{\prime }$, we have the formula:
  (10) $$\forall x_{1}\ldots \forall x_{k}\forall y_{1}\ldots \forall y_{k^{\prime }}Dep_{i}^{j}\left(x_{1},\ldots ,x_{k},y_{1},\ldots ,y_{k^{\prime }}\right)↔C_{F_{r_{i}}\left(x_{1},\ldots ,x_{k}\right)}^{r_{j}\left(y_{1},\ldots ,y_{k^{\prime }}\right)}.$$
  $$\forall x\forall yDep_{\mathrm{calls}}^{\mathrm{alarm}}\left(x,y\right)↔\exists z\mathrm{neighbor}\left(x,z\right)\wedge \left(z=y\right);\;\forall x\forall yDep_{\mathrm{alarm}}^{\mathrm{burglary}}\left(x,y\right)↔\left(x=y\right).$$

In the formula above, $C_{F_{r_{i}}\left(x_{1},\ldots ,x_{k}\right)}^{r_{j}\left(y_{1},\ldots ,y_{k^{\prime }}\right)}$ has free variables $x_{1},\ldots ,x_{k},y_{1},\ldots ,y_{k^{\prime }}$ and is built according to the four recursive rules that define $C_{F(t)}^{r^{\prime }\left(t^{\prime }\right)}$, replacing the tuples *t* and $t^{\prime }$ by *x* and *y*. We point out that such construction depends only on probability formulas in the relational Bayesian network Φ, and not on any *S*-structure. To build each $C_{F_{r_{i}}\left(x\right)}^{r_{j}\left(y\right)}$, one just starts from the probability formula $F_{r_{i}}\left(x\right)$ and follows the recursion rules until reaching the base cases, when $C_{F_{r_{i}}\left(x\right)}^{r_{j}\left(y\right)}$ will be formed by subformulas like ⊤,⊥, *S*-formulas φ(·) and equalities (·=·), possibly quantified on variables appearing in φ.

The relation Parent(·,·) is defined now over elements that represent ground atoms $r_{i}\left(t\right)$ and $r_{j}\left(t^{\prime }\right)$ such that $Dep_{i}^{j}\left(t,t^{\prime }\right)$, meaning that $r_{i}\left(t\right)⪯r_{j}\left(t^{\prime }\right)$. This can be achieved in two parts: ensuring that each $r_{i}\left(t\right)⪯r_{j}\left(t^{\prime }\right)$ implies $Parent(t,t^{\prime })$; and guaranteeing that $Parent(t,t^{\prime })$ is true only if $r_{i}\left(t\right)⪯r_{j}\left(t^{\prime }\right)$ for a pair of relations $r_{i},r_{j}$.

- For each pair $r_{i},r_{j}\in R$, with $a(r_{i})=k$ and $a\left(r_{j}\right)=k^{\prime }$, let *y* and $y^{\prime }$ denote $y_{1},\ldots ,y_{k}$ and $y_{1}^{\prime },\ldots ,y_{k^{\prime }}^{\prime }$, respectively:
  (11) $$\forall x\forall x^{\prime }\forall y_{1}\ldots \forall y_{k}\forall y_{1}^{\prime }\ldots \forall y_{k^{\prime }}^{\prime }R_{i}\left(x,y\right)\wedge R_{j}\left(x^{\prime },y^{\prime }\right)\wedge Dep_{i}^{j}\left(y,y^{\prime }\right)\to Parent\left(x,x^{\prime }\right).$$
  $$\forall x\forall x^{\prime }\forall y\forall y^{\prime }\mathrm{calls}\left(x\right)\wedge t_{1}\left(y,x\right)\wedge \mathrm{alarm}\left(x^{\prime }\right)\wedge t_{1}\left(y^{\prime },x\right)\wedge Dep_{\mathrm{calls}}^{\mathrm{alarm}}\left(y,y^{\prime }\right)\to Parent\left(x,x^{\prime }\right).$$
- Let $k=\max_{j}a\left(r_{j}\right)$ be the maximum arity in *R*, and let *y* and *y* denote the tuples $y_{1},\ldots ,y_{a(r_{i})}$ and $y_{1}^{\prime },\ldots ,y_{a(r_{j})}^{\prime }$, respectively:
  (12) $$\begin{array}{c} \forall x\forall x^{\prime }Parent\left(x,x^{\prime }\right)\to \exists y_{1}\ldots \exists y_{k}\exists y_{1}^{\prime }\ldots \exists y_{k}^{\prime }\underset{1\leq i,j\leq m}{⋁}R_{i}\left(x,y_{r_{i}}\right)\wedge R_{j}\left(x^{\prime },y_{r_{j}}^{\prime }\right)\wedge Dep_{i}^{j}\left(y_{r_{i}},y_{r_{j}}^{\prime }\right). \end{array}$$

**Definition** **4.** Given disjoint sets of relations S and R and a relational Bayesian network $\Phi =\{F_{r_{i}}|r_{i}\in R\}$, the formula $B_{\Phi }$ is the conjunction of all formulas in (2)–(12).

For some fixed relational Bayesian networks Φ, the formula $B_{\Phi }$ is satisfied only by *V*-structures D over a bipartite domain $D_{S}\cup D_{B}$ such that:

- the relations in *S* are interpreted in $D_{S}$, forming an *S*-structure $D_{S}$;
- there is a bijection *b* between the domain $D_{B}=D\setminus D_{S}$ and set of all ground *R*-atoms formed by the tuples in $D_{S}$;
- each $x\in D_{B}$ is linked exactly to one $r_{i}\in R$, via the predicate ${\bar{r}}_{i}\left(x\right)$, and exactly $k=a(r_{i})$ elements in $D_{S}$, via the relations $t_{1}\left(x,.\right),\ldots t_{k}\left(x,.\right)$, and no ground atom is represented through these links twice;
- the relation Parent(·,·) is interpreted as arcs in $D_{B}$ in such a way that $\langle D_{B},Parent\rangle$ form a directed graph that is the structure of the ground Bayesian network $B_{\Phi }\left(D_{S}\right)$.

### 3.2. Encoding Coherence via Acyclicity

The original formula $\psi_{\Phi }$ was intended to capture the coherence of the relational Bayesian network Φ. Our idea is to check the coherence by looking for cycles in the ground Bayesian network $B_{\Phi }\left(D_{S}\right)$ encoded in any *V*-structure satisfying $B_{\Phi }$. Hence, we replace $\psi_{\Phi }$ by an implication $B_{\Phi }\to \psi^{\prime }$, which is to be satisfied only by *V*-structures D such that, if D represents an *S*-structure $D_{S}$ and the resulting ground Bayesian network $B_{\Phi }\left(D_{S}\right)$, then $B_{\Phi }\left(D_{S}\right)$ is acyclic. Thus, $\psi^{\prime }$ should avoid cycles of the relation Parent in the *V*-structures satisfying it.

There is a cycle with Parent-arcs in a *V*-structure D over a domain *D* iff there exists a x∈D such that there is a path of Parent-arcs from *x* to itself. Consequently, detecting Parent-cycles reduces to computing Parent-paths or Parent-reachability. We say *y* is Parent-reachable from *x*, in a *V*-structure D, if there are $z_{0},\ldots ,z_{k}\in D$ such that $x=z_{0}$, $y=z_{k}$, and $D⊨{⋀}_{1\leq i\leq k}Parent\left(z_{i-1},z_{i}\right)$. Thus, for each *k*, we can define reachability through *k* Parent-arcs: $ParentPath_{k}\left(x,y\right)=\exists z_{0}\ldots \exists z_{k}\left(z_{0}=x\right)\wedge \left(z_{k}=y\right)\wedge {⋀}_{1\leq i\leq k}Parent\left(z_{i-1},z_{i}\right)$. Unfortunately, the size of the path (*k*) is unbounded *a priori*, as the domain *D* can be arbitrarily large. Therefore, there is no means in the first-order logic language to encode reachability, via arbitrarily large paths, with a finite number of formulas. In order to circumvent this situation, we can resort to a transitive closure logic.

Transitive closure logics enhance first-order logics with a transitive closure operator TC that we assume to be strict [25]. If φ(x,y) is a first-order formula, TC(φ)(x,y) means that *y* is φ-reachable from *x*, with a non-empty path. Accordingly, a *V*-structure D, over a domain *D*, satisfies TC(φ)(x,y) iff there is a k∈N and there are $z_{0},\ldots ,z_{k}\in D$ such that $x=z_{0}$, $y=z_{k}$ and $D⊨{⋀}_{1\leq i\leq k}\varphi \left(z_{i-1},z_{i}\right)$.

Employing the transitive closure operator, the existence of a Parent-path from a node *x* to itself (a cycle) is encoded directly by TC(Parent)(x,x); similarly, the absence of a Parent-cycle is enforced by $\psi^{\prime }=\forall x\neg \mathrm{TC}\left(Parent\right)\left(x,x\right)$.

At this point, the *V*-structures D over a domain *D* satisfying $B_{\Phi }\to \psi^{\prime }$ have the following format:

- either it encodes an *S*-structure in $D_{S}\subseteq D$ (the part of the domain satisfying $D_{S}\left(\cdot \right)$) and the corresponding *acyclic* ground Bayesian network $B_{\Phi }\left(D_{S}\right)$ in $D_{B}=D\setminus D_{S}$.
- or it is not the case that D encodes both an *S*-structure in $D_{S}\subseteq D$ and the corresponding ground Bayesian network $B_{\Phi }\left(D_{S}\right)$ in $D_{B}=D\setminus D_{S}$;

Back to the coherence-checking problem, we need to decide, for a fixed relational Bayesian network Φ, whether or not a given class S of *S*-structures ensures the acyclicity of the resulting ground Bayesian network $B_{\Phi }\left(D_{S}\right)$. Recall that the class S must be defined via a (first-order) *S*-formula $\theta_{S}$. As we are already employing the transitive closure operator in $\psi^{\prime }$, we can also allow its use in $\theta_{S}$, which is useful to express *S*-structures without cycles, for instance.

To check the coherence of Φ for a class S, we cannot just check the validity of:

(13) $$\theta_{S}\to \left(B_{\Phi }\to \psi^{\prime }\right),$$

because $\theta_{S}$ specifies *S*-structures over *D*, while $B_{\Phi }\to \psi^{\prime }$ presupposes that the *S*-structure is given only over $D_{S}=\left\{d\in D|D⊨D_{S}\left(d\right)\right\}⊊D$. To see the kind of problem that might occur, think of the class S of all *S*-structures D where each d∈D is such that $s_{i}\left(d\right)$ holds, for some unary predefined relation $s_{i}\in S$. Consider an *S*-structure D∈S ($D⊨\theta_{S}$), over a domain *D*. The formula $B_{\Phi }$ cannot be satisfied by D, for $D_{S}\left(x\right)$ must hold for all x∈D, because of the formulas in (9), so no x∈D can represent ground formulas, due to the formulas in (6), contradicting the restrictions in (7) that require all ground atoms to be represented. Hence, this D satisfies $\theta_{S}$ without encoding the ground Bayesian network, thus falsifying $B_{\Phi }$ and satisfying $B_{\Phi }\to \psi^{\prime }$, yielding the satisfaction of Formula (13). Consequently, Formula (13) is valid for this specific class S, no matter what the relational Bayesian network Φ looks like. Nonetheless, it is not hard to think of a Φ that is trivially incoherent for any class of *S*-structures, like $\Phi =\{F_{r}\left(x\right)=r\left(x\right)\}$, with S=⌀ and R={r}, where the probability formula associated with the relation r∈R is the indicator function r(x), yielding a cyclic dependency relation ⪯.

In order to address the aforementioned issue, we need to adapt $\theta_{S}$, constructing $\theta_{S}^{\prime }$ to represent the class S in the extended, bipartite domain $D=D_{S}\cup D_{B}$. The unary predicate $D_{S}\left(\cdot \right)$ is what delimits the portion of *D* that is dedicated to define the *S*-structure. Actually, we can define $D_{S}$ as the set $\{x\in D|D⊨D_{S}\left(x\right)\}\subseteq D$. Therefore, we must construct a *V*-formula $\theta_{S}^{\prime }$ such that the *V*-structure D satisfies $\theta_{S}^{\prime }$ iff the *S*-structure $D_{S}$, formed by $D_{S}\subseteq D$ and the interpretation of the *S* relations, satisfies $\theta_{S}$. That is, the *S*-formulas that hold in an *S*-structure $D^{\prime }\in S$ must hold for the subset of a *V*-structure D defined over the part of its domain that satisfies $D_{S}\left(\cdot \right)$. This can be performed by inserting *guards* in the quantifiers inside $\theta_{S}$.

**Definition** **5.** Given a (closed) *S*-formula $\theta_{S}$, $\theta_{S}^{\prime }$ is the formula resulting from applying the following substitutions to $\theta_{S}$:

- Replace each ∃xφ(x) in $\theta_{S}$ by $\exists xD_{S}\left(x\right)\wedge \varphi \left(x\right)$;
- Replace each ∀xφ(x) in $\theta_{S}$ by $\forall xD_{S}\left(x\right)\to \varphi \left(x\right)$.

Finally, we can define the formula that encodes the coherence of a relational Bayesian network Φ for a class of *S*-structures S:

**Definition** **6.** For disjoint sets of relations *S* and *R*, a given relational Bayesian network Φ and a class of *S*-structures defined by $\theta_{S}$, $C_{\Phi ,S}=\theta_{S}^{\prime }\to \left(B_{\Phi }\to \psi^{\prime }\right)$.

Putting all those arguments together, we obtain the translation of the coherence-checking problem to the validity of a formula from the transitive closure logic:

**Theorem** **1** (De Bona and Cozman [24])**.** *For disjoint sets of relations S and R, a given relational Bayesian network *Φ* and a class of S-structures S defined by $\theta_{S}$, *Φ* is coherent for S iff $C_{\Phi ,S}$ is valid.*

As first-order logic in general is already well-known to be undecidable, adding a transitive closure operator clearly does not make things easier. Nevertheless, decidability remains an open problem, even restricting the relations in *R* to be unary and assuming a decidable $\theta_{S}$ (even though there are some decidable fragments of first-order logic with transitive closure operators [25,26]). Similarly, a proof of general undecidability remains elusive.

### 3.3. A Weaker Form of Coherence

Jaeger introduced the coherence problem for RBNs as checking whether every input structure in a given class yields a probability distribution via an acyclic ground Bayesian network. Alternatively, we might define the coherence of an RBN as the existence of at least one input structure, out of a given class, resulting in an acyclic ground Bayesian network. This is closer to the satisfiability-like notion of coherence discussed by de Finetti and closer to work on probabilistic logic [27,28].

In this section, we show that, if one is interested in a logical encoding for this type of coherence for RBNs, the transitive closure operator can be dispensed with.

Suppose we have an RBN Φ and class S of input structures codified via a first-order formula $\theta_{S}$ and we want to decide whether Φ is coherent for some structure in S. This problem can be reduced to checking the satisfiability of a first-order formula, using the machinery introduced above, with the bipartite domain. This formula can be easily built as $\theta_{S}^{\prime }\wedge B_{\Phi }\wedge \psi^{\prime }$. By construction, this formula is satisfiable iff there is a structure D over a bipartite domain $D=D_{S}\cup D_{B}$ where $D_{S}$ encodes an *S*-structure in S ($D⊨\theta_{S}^{\prime }$), $D_{B}$ encodes the corresponding ground Bayesian network ($D⊨B_{\Phi }$) and the latter is acyclic ($D⊨\psi^{\prime }$). Nonetheless, since now we are interested in satisfiability instead of validity, we can replace $\psi^{\prime }$ by a formula $\psi^{\prime \prime }$ that does not employ the transitive closure operator.

The idea to construct $\psi^{\prime \prime }$ is to use a binary relation $Parent^{\prime }\left(\cdot ,\cdot \right)$ and to force it to extend, or to contain, the transitive closure of Parent(·,·). The formula $\psi^{\prime \prime }$ then also requires $Parent^{\prime }\left(\cdot ,\cdot \right)$ to be irreflexive. If there is such $Parent^{\prime }\left(\cdot ,\cdot \right)$, then Parent(·,·) must be acyclic. Conversely, if Parent(·,·) is acyclic, then $Parent^{\prime }\left(\cdot ,\cdot \right)$ can be interpreted as its transitive closure, being irreflexive. In other words, we want a structure to satisfy $\psi^{\prime \prime }$ iff it interprets a relation $Parent^{\prime }\left(\cdot ,\cdot \right)$ that both is irreflexive and extends the transitive closure of Parent(·,·).

In order to build $\psi^{\prime }$, the vocabulary *V* is augmented with the binary relation $Parent^{\prime }$. Now, we can define $\psi^{\prime \prime }$ as the conjunction of two parts:

- $\forall x\forall y\forall z\left(Parent\left(x,y\right)\to Parent^{\prime }\left(x,y\right)\right)\wedge \left(Parent^{\prime }\left(x,y\right)\wedge Parent^{\prime }\left(y,z\right)\to Parent^{\prime }\left(x,z\right)\right)$, forcing $Parent^{\prime }$ to extend the transitive closure of Parent;
- $\forall x\neg Parent^{\prime }\left(x,x\right)$, requiring $Parent^{\prime }$ to be irreflexive.

By construction, one can verify the following result:

**Theorem** **2.** For disjoint sets of relations S and R, a given relational Bayesian network *Φ* and a class of S-structures S defined by $\theta_{S}$, *Φ* is coherent for some structure in S iff $\theta_{S}^{\prime }\wedge B_{\Phi }\wedge \psi^{\prime \prime }$ is satisfiable.

The fact that $\theta_{S}^{\prime }\wedge B_{\Phi }\wedge \psi^{\prime \prime }$ does not use the transitive closure operator makes its satisfiability decidable for any decidable fragment of first-order logic.

## 4. Probabilistic Relational Models

In this section, we introduce the machinery of PRMs by following the terminology by Getoor et al. [19], focusing on the simple case where uncertainty is restricted to descriptive attributes, which are assumed to be binary. We also review the coherence problem for PRMs and the proposed solutions in the literature. In the next section, we show how this coherence problem can also be tackled via logic, as the coherence of RBNs.

### 4.1. Syntax and Semantics of PRMs

To define a PRM, illustrated in Example 5, we need a relational model, with classes associated with descriptive attributes and reference slots that behave like foreign keys. Intuitively, each object in a class is described by the values of its descriptive attributes, and reference slots link different objects. Formally, a relational schema is described by a set of classes $X=\{X_{1},\ldots ,X_{n}\}$, each of which associated with a set of *descriptive attributes* $A(X_{i})$ and a set of *reference slots* $R(X_{i})$. We assume descriptive attributes take values in {0,1}. A reference slot ρ in a class *X* (denoted X.ρ) is a reference to an object of the class Range[ρ] (its *range type*) specified in the schema. The *domain type* of ρ, Dom[ρ], is *X*. We can view this reference slot ρ as a function $f_{\rho }$ taking objects in Dom[ρ] and returning singletons of objects in Range[ρ]. That is, $f_{\rho }\left(x\right)=\left\{y\right\}$ is equivalent to x.ρ=y.

For any reference slot ρ, there is an *inverse slot* $\rho^{-1}$ such that $\mathrm{Range}\left[\rho^{-1}\right]=\mathrm{Dom}\left[\rho \right]$ and $\mathrm{Dom}\left[\rho^{-1}\right]=\mathrm{Range}\left[\rho \right]$. The corresponding function, $f_{\rho^{-}1}$ takes an object *x* from the class Range[ρ] and returns the set of objects $\left\{y|f_{\rho }\left(y\right)=\left\{x\right\}\right\}$ from the class Dom[ρ]. A sequence of slots (inverted or not) $K=\rho_{1},\ldots ,\rho_{k}$ is called a *slot chain* if $\mathrm{Range}\left[\rho_{i}\right]=\mathrm{Dom}\left[\rho_{i+1}\right]$ for all *i*. The function corresponding to a slot chain $K=\rho_{1},\rho_{2}$, $f_{K}$, is a type of composition of the functions $f_{\rho_{1}},f_{\rho_{2}}$, taking an object *x* from $\mathrm{Range}[\rho_{1}]$ and returning a set objects $\left\{z|\exists y:y\in f_{\rho_{1}}\left(x\right)\wedge z\in f_{\rho_{2}}\left(y\right)\right\}$ from $\mathrm{Range}[\rho_{2}]$. The corresponding function can be obtained by applying this type of composition two-by-two. We write y∈x.K when $y\in f_{K}\left(x\right)$.

An instance I of a relational schema populates the classes with objects, associating values with the descriptive attributes and reference slots. Formally, I is an interpretation specifying for each class X∈X: a set of objects I(X); a value A.x∈{0,1} for each descriptive attribute in A∈A(X) and each object x∈I(X); and an object x.ρ∈I(Range[ρ]) for each reference slot ρ∈R(X) and object x∈I(X). Note that, if x.ρ=y, $f_{\rho }\left(x\right)=\left\{y\right\}$. We use $I_{x.A}$ and $I_{x.\rho }$ to denote the value of x.A and x.ρ in I.

Given a relational schema, a PRM defines a probability distribution over its instances. In the simplest form, on which we focus, objects and the relations between them are given as input, and there is uncertainty only over the descriptive attributes values. A *relational skeleton* $\sigma^{r}$ is a partial specification of an instance that specifies a set of objects $\sigma^{r}\left(X_{i}\right)$ for each class $X_{i}$ in the schema besides the relation holding between these objects: ${\sigma^{r}}_{x.\rho }$ for each $x\in \sigma^{r}\left(X_{i}\right)$ and $\rho \in R(X_{i})$. A *completion* of a relational skeleton $\sigma^{r}$ is an instance I such that, for each class $X_{i}\in X$: $I\left(X_{i}\right)=\sigma^{r}\left(X_{i}\right)$ and, for each $x\in I(X_{i})$ and $\rho \in R(X_{i})$, $I_{x.\rho }={\sigma^{r}}_{x.\rho }$. We can see a PRM as a function taking relational skeletons and returning probability distributions over the completions of these partial instances, which can be seen as joint probability distributions for the random variables formed by the descriptive attributes of each object.

The format of a PRM resembles that of a Bayesian network: for each attribute X.A, we have a set of parents Pa(X.A) and the corresponding parameters P(X.A∣Pa(X.A)). The parent relation forms a direct graph, as usual, called the *dependency graph*; and the set of parameters define the conditional probability tables. The attributes in Pa(X.A) are called *formal parents*, as they will be instantiated for each object *x* in *X* according to the relational skeleton. There two types of formal parents: X.A can depend either on another attribute X.B of the same object or on an attribute X.K.B of other objects, where *K* is a slot chain.

In general, for an object *x*, x.K.B is a multiset {y.B∣y∈x.K}, whose size is defined by the relational skeleton. To compactly represent the conditional probability distribution when X.K.B∈Pa(X.A), the notion of *aggregation* is used. The attribute x.A will depend on some aggregate function γ of this multiset, like its mean value, mode, maximum or minimum, and so on; that is, γ(X.K.B) will be a formal parent of X.A.

**Definition** **7.** A *probabilistic Relational model* Π for a relational schema R is defined as a pair $\langle \Pi_{S},\Pi_{\theta }\rangle$ where:

- $\Pi_{S}$ defines, for each class X∈X and each descriptive attribute A∈A(X), a set of formal parents $\mathrm{Pa}\left(X.A\right)=\left\{U_{1},\ldots ,U_{l}\right\}$, where each $U_{i}$ has the form X.B or γ(X.K.B);
- $\Pi_{\theta }$ is the set of parameters defining legal Conditional Probability Distributions (CPDs) P(X.A∣Pa(X.A)) for each descriptive attribute A∈A(X) of each class X∈X.

The semantics of a PRM is given by the *ground* Bayesian network induced by a relational skeleton, where the descriptive attributes of each object are the random variables.

**Definition** **8.** A PRM $\Pi =\langle \Pi_{S},\Pi_{\theta }\rangle$ and a relational skeleton $\sigma^{r}$ define a ground Bayesian network where:

- There is a node representing each attribute x.A, for all $x\in \sigma^{r}\left(X_{i}\right)$, $A\in A(X_{i})$ and $X_{i}\in X$;
- For each $X_{i}\in X$, each $x\in \sigma^{r}\left(X_{i}\right)$ and each $A\in A(X_{i})$, there is a node representing γ(x.K.B) for each $\gamma \left(X_{i}.K.B\right)\in \mathrm{Pa}\left(X_{i}.A\right)$;
- Each x.A depends on parents x.B, for formal parents X.B∈Pa(X.A), and on parents γ(x.K.B), for formal parents γ(X.K.B)∈Pa(X.A), according to $\Pi_{S}$;
- Each γ(x.K.B) depends on parents y.B with y∈x.K;
- The CPD for P(x.A∣Pa(x.A)) is P(X.A∣Pa(X.A)), according to $\Pi_{\theta }$.
- The CPD for P(γ(x.K.B)∣Pa(γ(x.K.B))) is computed through the aggregation function γ.

The joint probability distribution over the descriptive attributes can be factored as usual to compute the probability of a specific instance I that is a completion of the skeleton $\sigma^{r}$. If we delete each γ(x.K.B) from the ground Bayesian network, making its children depend directly on the nodes y.B with y∈x.K (defining a new parent relation ${\mathrm{Pa}}^{\prime }$) and updating the CPDs accordingly, we can construct a *simplified* ground Bayesian network. The latter can be employed to factor the joint probability distribution over the descriptive attributes:

$$\begin{array}{cc} P(I|\sigma^{r},\Pi ) & =\prod_{x\in \sigma^{r}}\prod_{A\in A(x)}P\left(I_{x.A}|I_{{\mathrm{Pa}}^{\prime }\left(x.A\right)}\right)\\ & =\prod_{X_{i}}\prod_{x\in \sigma^{r}\left(X_{i}\right)}\prod_{A\in A(x)}P\left(I_{x.A}|I_{{\mathrm{Pa}}^{\prime }\left(x.A\right)}\right). \end{array}$$

Viewing Π as a function from skeletons to probability distributions over instances, we use $\Pi (\sigma^{r})$ to denote the probability distribution $P(I|\sigma^{r},\Pi )$ over the completions I of $\sigma^{r}$.

**Example** **5.** Recall again Scenario 4 in Example 2. We can define a PRM that returns the corresponding Bayesian network for each number and configuration of neighbors. In our relational schema, we have a class Person, whose set of descriptive attributes is A(Person)={burglary,alarm,calls}. Furthermore, to capture multiple neighbors, we also need a class Neighborhood, with two reference slots, R(Neighborhood)={neighbor1,neighbor2}, whose domain is Person. For instance, to denote that Alice and Bob are neighbors, we would have an object, say nAB, in the class Neighborhood, whose reference slots would be nAB.neighbor1=Alice and nAB.neighbor2=Bob.

We assume that the relation neighbor is reflexive (that is, for each Person x, there is always a Neighborhood$n_{x}$ with $n_{x}.\mathrm{neighbor}1=n_{x}.\mathrm{neighbor}2=x$) and symmetrical (if $x\in y.\mathrm{neighbor}1^{-1}.\mathrm{neighbor}2$, we also have $y\in x.\mathrm{neighbor}1^{-1}.\mathrm{neighbor}2$).

*For each descriptive attribute in our relational schema, we associate a set of formal parents and a conditional probability table, forming the following PRM* Π *to encode Scenario 4:*

- Pa(Person.burglary)=⌀; P(Person.burglary)=0.001;
- Pa(Person.alarm)={burglary}; P(Person.alarm∣burglary)=0.9 and P(Person.alarm∣¬burglary)=0.1;
- $\mathrm{Pa}\left(\mathrm{Person}.\mathrm{calls}\right)=\{\mathrm{or}\left(\mathrm{Person}.\mathrm{neighbor}1^{-1}.\mathrm{neighbor}2\right)\}$;
  $P(\mathrm{Person}.\mathrm{calls}|\mathrm{or}\left(\mathrm{Person}.\mathrm{neighbor}1^{-1}.\mathrm{neighbor}2\right)=c)=c$, for c∈{0,1}.

*Given a relational skeleton $\sigma^{r}$ with persons and neighbors,* Π *determines a joint probability distribution over the the descriptive attributes, via a Bayesian network. Consider a skeleton $\sigma^{r}$ with $\sigma^{r}\left(\mathrm{Person}\right)=\left\{x_{1},x_{2},x_{3}\right\}$ and $n12,n23\in \sigma^{r}\left(\mathrm{Neighborhood}\right)$, with ${\sigma^{r}}_{nij.\mathrm{neighbor}1}=x_{i}$ and ${\sigma^{r}}_{nij.\mathrm{neighbor}2}=x_{j}$, for each $nij\in \sigma^{r}\left(\mathrm{Neighborhood}\right)$, but such that no $n\in \sigma^{r}\left(\mathrm{Neighborhood}\right)$ has $n.\mathrm{neighbor}1=x_{1}$ and $n.\mathrm{neighbor}2=x_{3}$. Then, the resulting probability distribution is the model of Scenario 3 in Example 2, whose Bayesian network is given in Figure 2.*

### 4.2. Coherence via Colored Dependency Graphs

As with RBNs, for the model to be coherent, one needs to guarantee that the ground Bayesian network is acyclic. Getoor et al. [19] focused on guaranteeing that a PRM yields acyclic ground Bayesian networks for *all* possible relational skeletons. To achieve that, possible cycles are detected in the class dependency graph.

**Definition** **9.** Given a PRM Π, the *class dependency graph* $G_{\Pi }$ is a directed graph with a node for each descriptive attribute X.A and the following arcs:

- Type I arcs: 〈X.B,X.A〉, where X.B is a formal parent of X.A;
- Type II arcs: 〈Y.B,X.A〉, where γ(X.K.B) is a formal parent of X.A and Y=Range[X.K].

When the class dependency graph is acyclic, so is the ground Bayesian network for any relational skeleton. Nevertheless, it may be the case that, even for cyclic class dependency graphs, any relational skeleton occurring in practice leads to a coherent model. In other words, there might be classes of skeletons for which the PRM is coherent. To easily recognize some of these classes, Getoor et al. [19] put forward an approach based on identifying slot chains that are acyclic in practice. A set of slot chains $K_{ga}=\left\{K_{1},\ldots ,K_{m}\right\}$ is *guaranteed acyclic* if we are guaranteed that, for any possible relational skeleton $\sigma^{r}$, there is a partial ordering ⪯ over its objects such that, for each $K_{i}\in K_{ga}$, x≺y for any pair *x* and $y\in x.K_{i}$ (we use x≺y to denote x⪯y and x≠y).

**Definition** **10.** Given a PRM Π and a set of guaranteed acyclic slot chains $K_{ga}$, the *colored class dependency graph* $G_{\Pi }$ is a directed graph with a node for each descriptive attribute X.A and the following arcs:

- Yellow arcs: 〈X.B,X.A〉, where X.B is a formal parent of X.A;
- Green arcs: 〈Y.B,X.A〉, where γ(X.K.B) is a formal parent of X.A, Y=Range[X.K] and $K\in K_{ga}$;
- Red arcs: 〈Y.B,X.A〉, where γ(X.K.B) is a formal parent of X.A, Y=Range[X.K] and $K\notin K_{ga}$.

Intuitively, yellow cycles in the colored class dependency graph correspond to attributes of the same object, yielding a cycle in the ground Bayesian network. If we add some green arcs to such a cycle, then it is guaranteed that, departing from a node x.A in the ground Bayesian network, these arcs form a path to y.A, where x≺y, since ⪯ is transitive. Hence, *x* is different from *y*, and there is no cycle. If there is a red arc in a cycle, however, one may have a skeleton that produces a cycle.

A colored class dependency graph is *stratified* if every cycle contains at least one green arc and no red arc. Then:

**Theorem** **3** (Getoor et al. [19])**.** *Given a PRM* Π *and a set of guaranteed acyclic slot chains $K_{ga}$, if the colored class dependency graph $G_{\Pi }$ is stratified, then the ground Bayesian network is acyclic for any possible relational skeleton.*

In the result above and in the definition of guaranteed acyclic slot chains, “possible relational skeleton” refers to the class of skeletons that can occur in practice. The user must detect the guaranteed acyclic slot chains, taking advantage of his *a priori* knowledge on the possible skeletons in practice. For instance, consider a slot chain motherOf linking objects of the same class Person (Example 3). A genetic attribute, like Person.blueEyes, might depend on Person.motherOf.blueEyes. Mathematically, we can conceive of a skeleton with a cyclic relation motherOf, resulting in a red cycle in the colored class dependency graph. Nonetheless, being aware of the intended meaning of motherOf, we know that such skeletons are not possible in practice, so the cycle is green, and coherence is guaranteed.

Identifying guaranteed acyclic slot chains is by no means trivial. In fact, Getoor et al. [19] also define guaranteed acyclic (g.a.) reference slots and g.a. slot chains are defined as those formed only by g.a. reference slots. Still, these maneuvers miss the cases where two possible reference slots cannot be g.a. according to the same ⪯, but combine to form a g.a. slot chain. Getoor et al. [19] mention the possibility of assuming different partial orders to define different sets of g.a. slot chains: in that case, each ordering would correspond to a shade of green in the colored class dependency graph, and coherence would not be ensured if there were two shades of green in a cycle.

## 5. Logic-Based Approach to the Coherence of PRMs

The simplest approach to the coherence of PRMs, via the non-colored class dependency graph, is intrinsically incomplete, in the sense that some skeletons might yield a coherent ground Bayesian network even for cyclic graphs. The approach via colored class dependency graph allows some cyclic graphs (the stratified ones) to guarantee consistency for the class of all possible skeletons. However, this method depends on a pre-specified set of guaranteed acyclic slot chains, and the colored class dependency graph being stratified for this set is only a sufficient, not a necessary condition for coherence. Therefore, the colored class dependency graph method is incomplete, as well. Even using different sets of g.a. slot chains (corresponding to shades of green) to eventually capture all of them, it is still possible that a cycle with red arcs cannot entail incoherence in practice. Besides being incomplete, the graph-based method is not easily applicable to an arbitrary class of skeletons. Given a class of skeletons as input, the user would have to detect somehow which slot chains are guaranteed acyclic for that specific class; this can be considerably more difficult than ensuring acyclicity in the general case.

To address these issues, thus obtaining a general, complete method for checking the coherence of PRMs for a given class of skeletons, we can resort to the logic-based approach we introduced for the RBNs in previous sections. The goal of this section is to adapt the logic-basic techniques to PRMs.

PRMs can be viewed as RBNs, as conditional probability tables of the former can be embedded into combination functions of the latter. This translation is out of our scope though, and it suffices for our purposes to represent PRMs as random relational structures, taking *S*-structures to probability distributions on *R*-structures. While the *S*-vocabulary is used to specify classes of objects and relations between them (that is, the relational skeleton), the *R*-vocabulary expresses the descriptive attributes of the objects. Employing this logical encoding of PRMs, we can apply the approach from Section 3.1 to the coherence problem for PRMs.

To follow this strategy, we first show how a PRM can be seen as a random relational structure described by a logical language.

### 5.1. PRMs as Random Relational Structures

Consider a PRM $\Pi =\langle \Pi_{S},\Pi_{\theta }\rangle$ over a relational schema described by a set of classes $X=\{X_{1},\ldots X_{n}\}$, each associated with a set of descriptive attributes $A(X_{i})$ and a set of reference slots $R(X_{i})$. Given a skeleton $\sigma^{r}$, which is formed by objects and relations holding between them, the PRM Π yields a ground Bayesian network over the descriptive attributes of these objects, defining a probability distribution $\Pi (\sigma^{r})$ over the completions of $\sigma^{r}$. Hence, if the relational skeleton is given as a first-order *S*-structure over a set of objects and a set of unary relations *R* denotes their attributes, the PRM becomes a random relational structure.

We need to represent a skeleton $\sigma^{r}$ as a first-order *S*-structure Σ. Objects in $\sigma^{r}$ can be seen as the elements of the domain *D* of Σ. Note that PRMs are typed, with each object belonging to specific class $X_{i}\in X$. Thus, we use unary relations $X_{1},\ldots ,X_{n}$ in the vocabulary *S* to denote the class of each object. Accordingly, for each x∈D, $X_{i}\left(x\right)$ holds in Σ iff $x\in \sigma^{r}\left(X_{i}\right)$. As each object belongs to exactly one class in the relational skeleton, the class of possible first-order structures is restricted to those where the relations $X_{1},\ldots ,X_{n}$ form a partition of the domain.

The first-order *S*-structure Σ must also encode the relations holding between the objects in the skeleton that are specified via the values of the reference slots. To capture these, we assume they have unique names and consider, for each reference slot $X_{i}.\rho \in R\left(X_{i}\right)$ with $Range\left[\rho \right]=X_{j}$, a binary relation $S^{\rho }$. In Σ, $S^{\rho }\left(x,y\right)$ holds iff ${\sigma^{r}}_{x.\rho }=y$. Naturally, $S^{\rho }\left(x,y\right)$ should imply $X_{i}\left(x\right)$ and $X_{j}\left(y\right)$. Now, Σ encodes, through the vocabulary *S*, all objects of a given class, as well as the relations between them specified in the reference slots. In other words, there is a computable function $b_{S}$ from relational skeletons $\sigma^{r}$ to *S*-structures $\Sigma =b_{S}\left(\sigma^{r}\right)$. For $b_{S}$ to be a bijection, we make its codomain equal to its range.

The probabilistic vocabulary of the random relational structure corresponding to a PRM is formed by the descriptive attributes of every class in the relational schema. We assume that attributes in different classes have different names, as well, in order to define the vocabulary of unary relations $R=\{A\in A\left(X_{i}\right)|X_{i}\in X\}$. If $A_{j}$ is an attribute of $X_{i}$, $x.A_{j}=1$ (resp. $x.A_{j}=0$) in the PRM is mirrored by the ground *R*-atom $A_{j}\left(x\right)$ being true (resp. false) in the random relational structure. Thus, as a completion I corresponds to a value assignment to descriptive attributes of objects $x_{1},\ldots ,x_{m}$ from a relational skeleton $\sigma^{r}$, it also corresponds to an *R*-structure $D_{I}$ over a domain $D=\{x_{1},\ldots ,x_{m}\}$ in the following way: $D_{I}⊨A_{i}\left(x_{j}\right)$ iff $x_{j}.A_{i}=1$. Note that we assume that for $D_{I}$ to correspond to a completion I of $\sigma^{r}$, $D_{I}⊭A_{i}\left(x_{j}\right)$ whenever $A_{i}$ is not an attribute of the class X∈X such that $x_{j}\in \sigma^{r}\left(X\right)$. Let $b_{R}$ denote the function taking instances I and returning the corresponding *R*-structures $D_{I}=b_{R}\left(I\right)$. As we cannot recover the skeleton $\sigma^{r}$ from the *R*-structure $D_{I}=b_{R}\left(I\right)$, $b_{R}$ is not a bijection. Nevertheless, fixing a skeleton $\sigma^{r}$, there is a unique I such that $b_{R}\left(I\right)=D_{I}$.

Now, we can define a random relational structure $P_{\Pi }$ that corresponds to the PRM Π. For every relational skeleton $\sigma^{r}$ over a domain *D*, let $P_{\Pi }\left(b_{S}\left(\sigma^{r}\right)\right):{\mathrm{Mod}}_{R}\left(D\right)\to \left[0,1\right]$ be a probability distribution over *R*-structures such that $P_{\Pi }\left(b_{S}\left(\sigma^{r}\right)\right)\left(D_{R}\right)=\Pi \left(\sigma^{r}\right)\left(I_{R}\right)$, if $D_{R}=b_{R}\left(I_{R}\right)$, for a completion $I_{R}$ of $\sigma^{r}$, and $P_{\Pi }\left(b_{S}\right)\left(D_{R}\right)=0$ otherwise.

### 5.2. Encoding the Ground Bayesian Network and its Acyclicity

The probability distribution $P_{\Pi }\left(b_{S}\left(\sigma^{r}\right)\right)$ can be represented by a ground Bayesian network $B_{P_{\Pi }}\left(b_{S}\left(\sigma^{r}\right)\right)$, where nodes represent the ground *R*-atoms. The structure of this network is isomorphic to the simplified ground Bayesian network yielded by Π for the skeleton $\sigma^{r}$, if we ignore the isolated nodes representing the spurious $A_{i}\left(x_{j}\right)=0$, when $A_{i}$ is not an attribute of the class to which $x_{j}$ belongs. The coherence of $\Pi (\sigma^{r})$ depends on the acyclicity of the corresponding ground Bayesian network $B_{\Pi }\left(\sigma^{r}\right)$, which is acyclic iff $B_{P_{\Pi }}\left(\sigma^{r}\right)$ is so. Therefore, we can encode the coherence of a PRM Π for a skeleton $\sigma^{r}$ via the acyclicity of $B_{P_{\Pi }}\left(b_{s}\left(\sigma^{r}\right)\right)$ by applying the techniques from Section 3.

We want to construct a formula that is satisfied only by those *S*-structures $b_{S}\left(\sigma^{r}\right)$ such that $\Pi (\sigma^{r})$ is coherent. Again, we consider an extended, bipartite domain $D=D_{S}\cup D_{B}$, with $b_{S}\left(\sigma^{r}\right)$ encoded over $D_{S}$ and the structure of $B_{P_{\Pi }}\left(\sigma^{r}\right)$ encoded in $D_{B}$. We want to build a formula $B_{\Pi }$ that is satisfied by structures D over $D=D_{S}\cup D_{B}$ such that, if D encodes $b_{S}\left(\sigma^{r}\right)$ over $D_{S}$, then D encodes the structure of $B_{P_{\Pi }}\left(b_{S}\left(\sigma^{r}\right)\right)$ over $D_{B}$. The nodes are encoded exactly as shown in Section 3.1.

To encode the arcs, we employ once more a relation Parent(·,·). $Parent(y,y^{\prime })$ must hold only if $x,y\in D_{B}$ denote ground *R*-atoms $A_{i}\left(x\right)$ and $A_{j}\left(x^{\prime }\right)$ such that $x^{\prime }.A_{j}\in {\mathrm{Pa}}^{\prime }\left(x.A_{i}\right)$ in the simplified ground Bayesian network, which is captured by the formula $Dep_{i}^{j}\left(x,x^{\prime }\right)$, as in Section 3.1. The only difference here is that now $Dep_{i}^{j}\left(x,x^{\prime }\right)$ can be defined directly. We use $Dep_{i}^{j}\left(x,x^{\prime }\right)$ here to denote a formula recursively defined, not an atom over the binary relation $Dep_{i}^{j}\left(\cdot ,\cdot \right)$. For each pair $A_{i},A_{j}\in R$, we can simply look at $\Pi_{S}$ to see the conditions on which $x^{\prime }.A_{j}$ is a parent of $x.A_{i}$ in the simplified ground Bayesian network ($x^{\prime }.A_{j}\in {\mathrm{Pa}}^{\prime }\left(x.A_{i}\right)$), in which case $A_{j}\left(x^{\prime }\right)$ will be a parent of $A_{i}\left(x\right)$ in $B_{P_{\pi }}\left(b_{S}\left(\sigma^{r}\right)\right)$. If $X.A_{j}\in \mathrm{Pa}\left(X.A_{i}\right)$, then $Dep_{i}^{j}\left(x,x^{\prime }\right)$ should be true whenever $x=x^{\prime }$. If $\gamma \left(X.K.A_{j}\right)\in \mathrm{Pa}\left(X.A_{i}\right)$, for a slot chain *K*, then $x.K.A_{j}\subseteq {\mathrm{Pa}}^{\prime }\left(x.A_{i}\right)$ and $Dep_{i}^{j}\left(x,x^{\prime }\right)$ should be true whenever $x^{\prime }$ is related to *x* via $K=\rho_{1},\ldots ,\rho_{k}$. This is the case if:

$$\exists y_{1},\exists y_{2}\ldots \exists y_{k-1}S^{\rho_{1}}\left(x,y_{1}\right)\wedge S^{\rho_{2}}\left(y_{1},y_{2}\right)\wedge \ldots \wedge S^{\rho_{k}}\left(y_{k-1},x^{\prime }\right)$$

is true. If K=ρ, this formula is simply $S^{\rho }\left(x,x^{\prime }\right)$.

Note that it is possible that both $X.A_{j}$ and $\gamma (X.K.A_{j})$ are formal parents of $X.A_{i}$, and there can even be different parents $\gamma (X.K.A_{j})$, for different *K*. Thus, we define $Dep_{i}^{j}\left(x,x^{\prime }\right)$ algorithmically. Initially, make $Dep_{i}^{j}\left(x,x^{\prime }\right)=⊥$. If $X.A_{j}\in \mathrm{Pa}\left(X.A_{i}\right)$ for some $A_{j}$, make $Dep_{i}^{j}\left(x,x^{\prime }\right)=Dep_{i}^{j}\left(x,x^{\prime }\right)\vee \left(x=x^{\prime }\right)$. Finally, for each $\gamma (X.K.A_{j})$ in $\mathrm{Pa}(X.A_{i})$, for a slot chain $K=\rho_{1},\ldots ,\rho_{k}$, make:

$$Dep_{i}^{j}\left(x,x^{\prime }\right)=Dep_{i}^{j}\left(x,x^{\prime }\right)\vee \exists y_{1},\exists y_{2}\ldots \exists y_{k-1}S^{\rho_{1}}\left(x,y_{1}\right)\wedge S^{\rho_{2}}\left(y_{1},y_{2}\right)\wedge \ldots \wedge S^{\rho_{k}}\left(y_{k-1},x^{\prime }\right),$$

using fresh $y_{1},\ldots ,y_{k-1}$.

Analogously to Section 3.1, we have a formula $B_{\Pi }$, for a fixed PRM Π, that is satisfied only by structures D over a bipartite domain $D_{S}\cup D_{B}$ where the Parent(·,·) relation over $D_{B}$ brings the structure of the ground Bayesian network $B_{P_{\Pi }}\left(\Sigma \right)$ corresponding to the *S*-structure Σ encoded in $D_{S}$. Again, acyclicity can be captured via a transitive closure operator: $\psi_{\Pi }=\forall x\neg \mathrm{TC}\left(Parent\right)\left(x,x\right)$. The PRM Π is coherent for a skeleton $\sigma^{r}$ if, for every structure D over a bipartite domain $D_{S}\cup D_{B}$ encoding $b_{S}\left(\sigma^{r}\right)$ in $D_{S}$, we have $D⊨B_{\Pi }\to \psi_{\Phi }$.

Consider now a class S of skeletons $\sigma^{r}$ such that $\left\{b_{S}\left(\sigma^{r}\right)|\sigma^{r}\in S\right\}$ is the set of *S*-structures satisfying a first-order formula $\theta_{S}$. To check whether the PRM Π is coherent for the class S, we construct $\theta_{S}^{\prime }$ by inserting guards to the quantifiers, as explained in Definition 5. Finally, the PRM Π is coherent for a class S of relational skeletons iff $\theta_{S}^{\prime }\to \left(B_{\Pi }\to \psi_{\Pi }\right)$ is valid.

We have thus succeeded in turning coherence checking for PRMs into a logical inference, by adapting techniques we developed for RBNs. In the next section, we travel, in a sense, the reverse route: we show how to adapt the existing graph-based techniques for coherence checking of PRMs to coherence checking of RBNs.

## 6. Graph-Based Approach to the Coherence of RBNs

The logic-based approach to the coherence problem for RBNs can be applied to an arbitrary class of input structures, as long as the class can be described by a first-order formula, possibly with a transitive closure operator. Given any class of input structures S, via the formula $\theta_{S}$, we can verify the coherence of a RBN Φ via the validity of $\theta_{S}^{\prime }\to \left(B_{\Phi }\to \psi^{\prime }\right)$, as explained in Section 3. Furthermore, this method is complete, as Φ is coherent for S if and *only if* such a formula is valid. Nonetheless, completeness and flexibility regarding the input class come at a very high price, as deciding the validity of this first-order formula involving a transitive closure operator may be computationally hard, if at all decidable. Therefore, RBNs users can benefit from the ideas introduced by Getoor et al. [19] for the coherence of PRMs, using the (colored) dependency graphs. While Jaeger [23] proposes to investigate the coherence for a given class of models described by a logical formula, Getoor et al. [19] are interested in a single class of inputs: the skeletons that are possible in practice. With *a priori* knowledge, the RBN user perhaps can attest to the acyclicity of the resulting ground Bayesian network for all possible inputs.

Any arc $\langle r^{\prime }\left(t^{\prime }\right),r\left(t\right)\rangle$ in the output ground Bayesian network $B_{\Phi }\left(D_{S}\right)$, for an RBN Φ and input $D_{S}$ reflects that the probability formula $F_{r}\left(x\right)$, when x=t, depends on $r(t^{\prime })$. Hence, possible arcs in this network can be anticipated by looking into the probability formulas $F_{r}\left(x\right)$, for the probabilistic relations r∈R, in the definition of Φ. In other words, by inspecting the probability formula $F_{r}\left(x\right)$, we can detect those $r^{\prime }\in R$ for which an arc $\langle r^{\prime }\left(t^{\prime }\right),r\left(t\right)\rangle$ can possibly occur in the ground Bayesian network. Similarly to the class dependency graph for PRMs, we can construct a high-level dependency graph for RBNs that brings the possible arcs, and thus cycles, in the ground Bayesian network.

**Definition** **11.** Given an RBN Φ, the *R-dependency graph* $G_{\Phi }$ is a directed graph with a node for each probabilistic relation r∈R and the following arcs:

- Type I arcs: $\langle r^{\prime },r\rangle$, where $r^{\prime }\left(x\right)$ occurs in $F_{r}\left(x\right)$ outside the scope of a combination function;
- Type II arcs: $\langle r^{\prime },r\rangle$, where $r^{\prime }\left(y\right)$ occurs in $F_{r}\left(x\right)$ inside the scope of a combination function.

Intuitively, a Type I arc $\langle r^{\prime },r\rangle$ in the *R*-dependency graph of an RBN Φ means that, for any input structure $D_{S}$ over *D* and any tuple $t\in D^{a(r)}$, $F_{r}\left(t\right)$ depends on $r^{\prime }\left(t\right)$ in the ground Bayesian network $B_{\Phi }\left(D_{S}\right)$; formally, $r^{\prime }\left(t\right)\in \alpha \left(F_{r}\left(x\right),t,D_{S}\right)$. For instance, if $F_{r_{1}}\left(x\right)=\mathrm{mean}(\{\;|r_{2}\left(y\right)|y;S\left(x,y\right)\left|\;\}\right)\left(1-r_{3}\left(x\right)\right)$, then, given any *S*-structure, $F_{r_{1}}\left(t\right)$ depends on $r_{3}\left(t\right)$ for any *t*. Type II arcs capture dependencies that are contingent on the *S*-relations holding in the input structure. In other words, a Type II arc $\langle r^{\prime },r\rangle$ means that $F_{r}\left(t\right)$ will depend on $r^{\prime }\left(t^{\prime }\right)$ if some *S*-formula φ holds in the input structure $D_{S}$; $D_{S}⊨\varphi$. For instance, if $F_{r_{1}}\left(x\right)=\left(\mathrm{mean}(\{\;\right|r_{2}\left(y\right)|y;S\left(x,y\right)\left|\;\}\right)\left(1-r_{3}\left(x\right)\right)$, $r_{1}\left(t\right)$ depends on $r_{2}\left(t^{\prime }\right)$ (for $t,t^{\prime }$ in the domain *D*) in the output ground Bayesian network iff the input $D_{S}$ is such that $D_{S}⊨S\left(t,t^{\prime }\right)$. If combination functions are nested, the corresponding *S*-formula might be fairly complicated. Nevertheless, the point here is simply noting that, given a Type II arc $\langle r^{\prime },r\rangle$, the conditions on which r(t) is actually a child of $r^{\prime }\left(t\right)$ in the ground Bayesian network can be expressed with an *S*-formula parametrized by $t,t^{\prime }$, which will be denoted by $\varphi_{S}^{r,r^{\prime }}\left(t,t^{\prime }\right)$. Consequently, for $t,t^{\prime }\in D$, $D_{S}⊨\varphi_{S}^{r,r^{\prime }}\left(t,t^{\prime }\right)$ iff $r^{\prime }\left(t^{\prime }\right)\in \alpha \left(F_{r}\left(x\right),t,D_{S}\right)$, i.e., r(t) depends on $r^{\prime }\left(t^{\prime }\right)$ in $B_{\Phi }\left(D_{S}\right)$.

As each arc in the ground Bayesian network corresponds to an arc on the *R*-dependency graph, when the latter is acyclic, so will be the former, for any input structure. As it happens with class dependency graphs and PRMs, though, a cycle in the *R*-dependency graph does not entail a cycle in the ground Bayesian network if a Type II arc is involved. It might well be the case that the input structures $D_{S}$ found in practice do not cause cycles to occur. This can be captured via a colored version of the *R*-dependency graph.

In the same way that Type I arcs in the class dependency graph of a PRM relate to attributes of different objects, in the *R*-dependency graph of an RBN, these arcs encode the dependency between relations $r,r^{\prime }\in R$ to be grounded with (possibly) different tuples. For a PRM, the ground Bayesian network can never reflect a cycle with green arcs, but no red one, in the class dependency graph, for a sequence of green arcs guarantees different objects, according to a partial ordering. Analogously, with domain knowledge, the user can identify Type II arcs in the *R*-dependency graph whose sequence will prevent cycles in the ground Bayesian network, via a partial ordering over the tuples.

For a vocabulary *S* of predefined relations, let $T^{D}=⋃\left\{D^{a}|a\in N\right\}$ denote the set of all tuples with elements of *D*. We say a set $A_{ga}=\left\{\langle r_{i}^{\prime },r_{i}\rangle |1\leq i\leq n\right\}$ of Type II arcs is guaranteed acyclic if, for any possible input structure $D_{S}$ over *D*, there is a partial ordering ⪯ over $T^{D}$ such that, if $D_{S}⊨\varphi_{S}^{r,r^{\prime }}\left(t,t^{\prime }\right)$ for some $t,t^{\prime }\in T^{D}$, then $t≺t^{\prime }$. Here, again, “possible” means “possible in practice”.

**Definition** **12.** Given the *R*-dependency graph of an RBN Φ and a set $A_{ga}$ of guaranteed acyclic type II arcs, the *colored R-dependency graph* $G_{\Pi }$ is a directed graph with a node for each r∈R and the followin arcs:

- Yellow arcs: Type I arcs in the *R*-dependency graph;
- Green arcs: Type II arcs $\langle r^{\prime },r\rangle$ in the *R*-dependency graph such that $\langle r^{\prime },r\rangle \in A_{ga}$;
- Red arcs: The remaining (Type II) arcs in the *R*-dependency graph.

Again, yellow cycles in the colored *R*-dependency graph correspond to relations r∈R grounded with the same tuple *t*, yielding a cycle in the ground Bayesian network. If green arcs are added to a cycle, then it is guaranteed that, departing from a node r(t) in the ground Bayesian network, these arcs form a path to $r(t^{\prime })$, where $t≺t^{\prime }$ for a partial ordering ⪯, and there is no cycle. Once more, red arcs in cycles may cause $t=t^{\prime }$, and coherence is not ensured. Calling *stratified* a *R*-dependency graph whose every cycle contains at least one green arc and no red arc, we have:

**Theorem** **4.** Given the R-dependency graph of an RBN *Φ* and a set $A_{ga}$ of guaranteed acyclic Type II arcs, if the colored class dependency graph $G_{\Phi }$ is stratified, then the ground Bayesian network is acyclic for any possible input structure.

Of course, detecting guaranteed acyclic Type II arcs in *R*-dependency graphs of RBNs is even harder than, as a generalization of, detecting guaranteed acyclic slot chains in PRMs. In any case, if the involved relations $r,r^{\prime }\in R$ are unary, one is in a position similar to finding acyclic slot chains, as the arguments of $r,r^{\prime }$ can be seen as objects, and only a partial ordering over the elements of the domain (not tuples) is needed.

## 7. Conclusions

In this paper, we examined a new version of *coherence checking*, a central problem in the foundations of probability as conceived by de Finetti. The simplest formulation of coherence checking takes a set of events and their probabilities and asks whether there can be a probability measure over an appropriate sample space [1]. This sort of problem is akin to inference in propositional probabilistic logic [28]. Unsurprisingly, similar inference problems have been studied in connection with first-order probabilistic logic [27]. Our focus here is on coherence checking when one has events specified by first-order expressions, on top of which one has probability values and independence relations. Due to the hopeless complexity of handling coherence checking for any possible set of assessments and independence judgments, we focus on those specifications that enhance the popular language of Bayesian networks. In doing so, we address a coherence checking problem that was discussed in the pioneering work by Jaeger [23].

We have first examined the problem of checking the coherence of relational Bayesian networks for a given class of input structures. We used first-order logic to encode the output ground Bayesian network into a first-order structure, and we employed a transitive closure operator to express the acyclicity demanded by coherence, finally reducing the coherence checking problem to that of deciding the validity of a logical formula. We conjecture that Jaeger’s original proposal concerning the format of the formula encoding the consistency of a relational Bayesian network Φ for a class *S* cannot be followed as originally stated; as we have argued, the possible number of tuples built from a domain typically outnumbers its size, so that there is no straightforward way to encode the ground Bayesian network, whose nodes are ground atoms, into the input *S*-structure. Therefore, it is hard to think of a method that translates the acyclicity of the ground Bayesian network into a formula $\varphi_{\Phi }$ to be evaluated over an input structure in the class *S* (satisfying $\theta_{S}$). Our contribution here is to present a logical scheme that bypasses such difficulties by employing a bipartite domain, encoding both the *S*-structure and the corresponding Bayesian network. We have also extended those results to PRMs, in fact mixing the existing graph-based techniques for coherence checking with our logic-based approach. Our results seem to be the most complete ones in the literature.

Future work includes searching for decidable instances of the formula encoding the consistency of a relational Bayesian network for a class of input structures and exploring new applications for the logic techniques herein developed.

## Abbreviations

The following abbreviations are used in this manuscript:

CPD	Conditional Probability Table
g.a.	guaranteed acyclic
iff	if and only if
PRM	Probabilistic Relational Model
PSAT	Probabilistic Satisfiability
RBN	Relational Bayesian Network
